# Optimization of the antimicrobial peptide Bac7 by deep mutational scanning

**DOI:** 10.1186/s12915-022-01304-4

**Published:** 2022-05-16

**Authors:** Philipp Koch, Steven Schmitt, Alexander Heynisch, Anja Gumpinger, Irene Wüthrich, Marina Gysin, Dimitri Shcherbakov, Sven N. Hobbie, Sven Panke, Martin Held

**Affiliations:** 1 Bioprocess Laboratory, Department of Biosystems Science and Engineering, ETH Zurich, Basel, Switzerland; 2 Machine Learning and Computational Biology, Department of Biosystems Science and Engineering, ETH Zurich, Basel, Switzerland; 3 Institute of Medical Microbiology, University of Zurich, Zurich, Switzerland

**Keywords:** Antibiotics, Antimicrobial peptides, Drug discovery, High-throughput screening, Antimicrobials, Deep mutational scanning, Proline-rich antimicrobial peptides, Protein synthesis inhibitor, Antimicrobial resistance, Sequence-activity relationship

## Abstract

**Background:**

Intracellularly active antimicrobial peptides are promising candidates for the development of antibiotics for human applications. However, drug development using peptides is challenging as, owing to their large size, an enormous sequence space is spanned. We built a high-throughput platform that incorporates rapid investigation of the sequence-activity relationship of peptides and enables rational optimization of their antimicrobial activity. The platform is based on deep mutational scanning of DNA-encoded peptides and employs highly parallelized bacterial self-screening coupled to next-generation sequencing as a readout for their antimicrobial activity. As a target, we used Bac7_1-23_, a 23 amino acid residues long variant of bactenecin-7, a potent translational inhibitor and one of the best researched proline-rich antimicrobial peptides.

**Results:**

Using the platform, we simultaneously determined the antimicrobial activity of >600,000 Bac7_1-23_ variants and explored their sequence-activity relationship. This dataset guided the design of a focused library of ~160,000 variants and the identification of a lead candidate Bac7PS. Bac7PS showed high activity against multidrug-resistant clinical isolates of *E. coli,* and its activity was less dependent on SbmA, a transporter commonly used by proline-rich antimicrobial peptides to reach the cytosol and then inhibit translation. Furthermore, Bac7PS displayed strong ribosomal inhibition and low toxicity against eukaryotic cells and demonstrated good efficacy in a murine septicemia model induced by *E. coli*.

**Conclusion:**

We demonstrated that the presented platform can be used to establish the sequence-activity relationship of antimicrobial peptides, and showed its usefulness for hit-to-lead identification and optimization of antimicrobial drug candidates.

**Supplementary Information:**

The online version contains supplementary material available at 10.1186/s12915-022-01304-4.

## Background

The drug development field is in urgent need of novel compounds to deliver the next generation of antibiotics to combat multidrug-resistant (MDR) bacteria [1]. Several promising leads have been identified in the group of antimicrobial peptides (AMPs), some of which are currently in drug development pipelines [2]. However, failure rates in clinical trials [3] are high, often because a majority of these molecules form pores in bacterial membranes or disintegrate them completly, a mode of action (MoA) that is prone to cause toxicity against human cells [4].

Proline-rich AMPs usually do not lyse but are interfering with the activity of intracellular targets essential for survival such as ribosomes [5]. One of the most intensively researched proline-rich AMPs is bactenecin-7 (Bac7). It is a 60 amino acid long linear peptide that was first isolated from bovine neutrophils [6]. In vitro studies on Bac7 truncates indicate that the two N-terminal arginine residues are needed for efficient uptake [7] and its C-terminus can be truncated resulting in peptides with a length of 35, 23, and 16 amino acids at only a minor loss of antimicrobial activity [8]. Moreover, the antimicrobial activity can be increased via modulation of the amino acid sequence [7, 9, 10]. To interact with the ribosome and inhibit protein translation, Bac7 crosses the outer membrane of Gram-negative bacteria via not yet fully elucidated mechanisms and then traverses the inner membrane through the SbmA transporter [11]. Bac7 displays high activity against many species of the Gram-negative Enterobacteriaceae [8, 12], a family of bacteria in which *sbmA *is expressed, and which are currently listed as “critical priority pathogens” by the WHO [13]. Research on Bac7 may thus offer a path towards developing a treatment against these threatening pathogens.

The standard method to study the sequence-activity relationship of AMPs relies on the chemical synthesis of mildly modulated peptides followed by activity tests using antimicrobial susceptibility assays. Due to limited throughput and costs of peptide synthesis, these studies can typically deliver only very few data points [9, 14].

To expand the coverage of protein or peptide sequence-activity relationships in general, deep mutational scanning (DMS) methods are widely used as they grant access to millions of variants and data points in single experiments [15]. Most frequently, these methods are used to study the effects of single amino acid residue substitutions [16], since introducing multiple substitutions leads to a combinatorial explosion of possible variants. In DMS, first, large libraries are produced by systematically varying the coding DNA sequence by chemical DNA synthesis methods or error-prone polymerase chain reaction (epPCR) [17]. The resulting peptide or protein variants are then expressed recombinantly in cells and subjected to screening or selection protocols, allowing the correlation of phenotype of the variants with a measurable output, e.g., cell survival or the level to which genetically encoded fluorophores are synthesized. To also ascertain the genotype, next-generation sequencing (NGS) is used to identify and quantify the encoding DNA fragments [18]. In the case of AMPs, DMS can be based on self-screening where the degree to which growth is affected by AMPs is estimated via quantification of the relative abundance of peptide-encoding DNA fragments [19]. Recently, DMS studies investigated the effect of amino acid residue substitutions of the proline-rich AMP apidaecin and oncocin on critical interactions with the ribosome [20, 21]. However, DMS has never been exploited as a platform for hit-to-lead optimization of antimicrobials. If applied successfully, the coverage of the AMP sequence-activity relationships could be increased considerably and deliver valuable clues for the design of AMP drug candidates.

In this study, we optimized the 23 amino acid truncate of Bac7 (Bac7_1-23_) in two DMS rounds. In the first round, we screened a Bac7_1-23_ library consisting of 601,551 randomly mutagenized variants and assessed their growth inhibitory effects when expressed intracellularly in *E. coli*. This enabled us to determine the contribution of each amino acid residue substitution to antimicrobial activity. Guided by these results, we performed a second DMS round with a focused, semi-rationally designed library of Bac7_1-23_ covering 156,779 variants. After assessing the effect on growth inhibition of each variant, we were able to build a peptide bearing the most activity-enhancing amino acid residue combination. This new-to-nature peptide called Bac7PS has a higher activity towards a broad panel of bacterial pathogens than Bac7_1-23_, low toxicity against eukaryotic cells, and good efficacy in vivo studies.

## Results

### Deep mutational scanning of Bac7_1-23_ using random mutagenesis

**Fig. 1:**
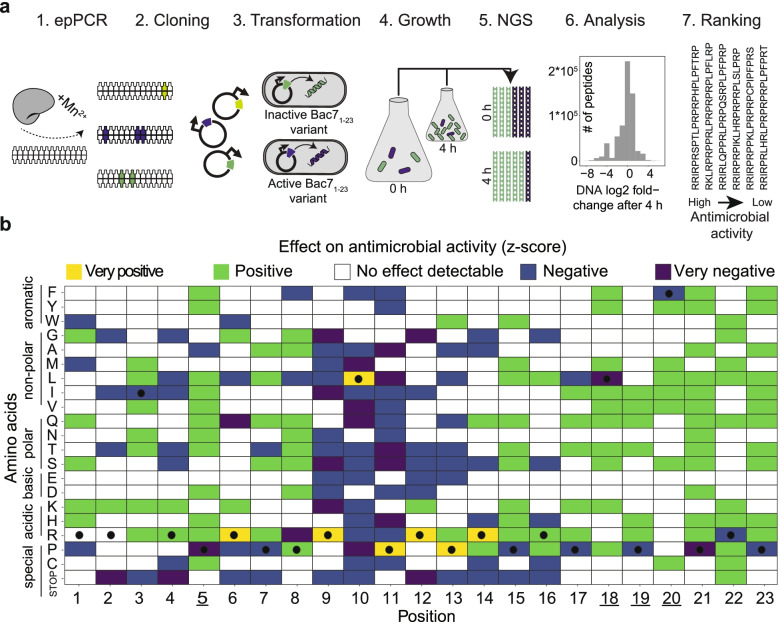
Sequence-activity relationship of Bac7_1-23_. **a** DMS workflow. epPCR: the Bac7_1-23_ gene is amplified at a high error rate using an error-prone DNA polymerase in the presence of Mn^2+^. Cloning: the mutagenized DNA sequences are inserted into plasmids downstream of inducible promoters. Transformation: *E. coli* TOP10 is transformed with the generated peptide-encoding DNA library. Growth: the pooled transformants are grown in a single shaking flask (*n* = 3), peptide synthesis is induced and plasmids are isolated after 4 h. NGS: the abundance of each peptide-encoding DNA sequence is determined by NGS at the time of induction and 4 h later. Analysis: for each peptide-encoding DNA sequence, the log2-fold change is determined (log2 ratio of abundances at the two time points). Histogram showing the log2-fold changes of the abundance of the peptide-encoding DNA of all 601,551 variants. Ranking: peptide sequences are ranked by the degree of the observed antimicrobial effect. The more negative a log2-fold change, the higher the observed antimicrobial effect and vice versa. **b** Bac7_1-23_ sequence-activity relationship displaying the magnitude of the observed antimicrobial effect. For each amino acid residue substitution (and stop codon), the enrichment in higher or lower antimicrobial peptides is determined and a *z*-score (z) is calculated (see the “Methods” section). *z* corresponds to the number of standard deviations by which the calculated enrichment lies above (positive values) or below (negative values) the mean a null distribution indicating no enrichment. *z* is empirically divided into four groups, corresponding to very positive (yellow; z ≥ 40), positive (green; z ≥ 4), negative (blue; z ≤ -4), or very negative (purple; *z* ≤ −40) effects on the antimicrobial activity. No effect on growth inhibition is detectable if the *z* is close to 0 (white; −4 < *z* < 4). Black dots are used for indication of Bac7_1-23_ wild-type amino acid residues. The underlined positions are chosen as targets for the subsequent site-saturation mutagenesis.

First, we performed DMS of Bac7_1-23_ to identify all amino acid residues essential for antimicrobial activity and those amendable to further activity optimization. To do so, the Bac7_1-23_ coding gene was randomly mutagenized by epPCR, and the modified DNA fragments were ligated into plasmids allowing their expression from the tightly regulated P_BAD_ promotor (Fig. 1a). To determine the antimicrobial activity of the synthesized peptide variants, we transformed *E. coli* TOP10 and induced their intracellular synthesis. Growth curves recorded for 94 randomly selected strains in microtiter plates indicated that about half of the Bac7_1-23_ variants efficiently suppressed the growth of the respective host (Additional file 1: Fig. S1). To assess the antimicrobial effect of a much larger proportion of the randomly mutated library at once, we grew around 500 million transformed *E. coli* TOP10 cells expressing the entire peptide-encoding library in a shake flask (*n* = 3; Additional file 1: Fig. S2), induced peptide synthesis, and counted the abundance of each peptide-encoding DNA sequence at the time of induction and 4 h later by NGS (Additional file 2). As the synthesis of active peptides limits the growth of their hosts and the propagation of the peptide-encoding DNA [19], a reduction of the relative abundance of the peptide-encoding DNA of each variant, expressed as log2-fold change, is representative of antimicrobial activity (Fig. 1a). In total, the library consisted of 601,551 different Bac7_1-23_ variants, ranked from the most active (=lowest negative log2-fold change) to the least active peptide (=highest positive log2-fold change) (Additional file 3). Among more heavily substituted variants (Additional file 1: Fig. S3), we found 398 peptides with one amino acid substitutions to Bac7_1-23_ (87% out of 460 possible variants), 21,567 double substitutions (21% out of 101,200 possible variants), 185,993 triple substitutions (1.3% out of ~14 million possible variants), and 228,433 quadruple substitutions (0.01% out of ~1.4 billion possible variants).

### Sequence-activity relationship of Bac7_1-23_

To determine the effect of each amino acid residue on antimicrobial activity, we investigated the ranking of 601,551 Bac7_1-23_ variants (Additional file 3). As NGS-based abundancy rankings can be error-prone, especially at low DNA fragment read counts [22], here ranking should be considered a qualitative instead of a quantitative measure.

**Fig. 2:**
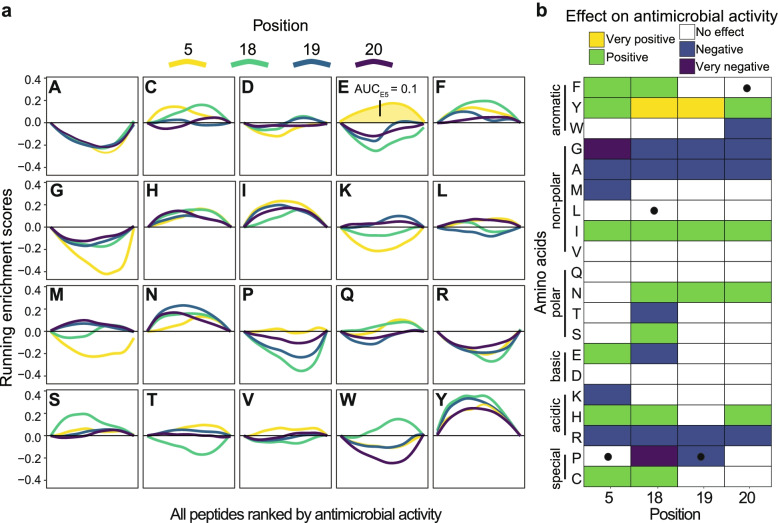
DMS of Bac7_1-23_ site-saturation mutagenesis library. **a** Enrichment curves. Peptides are first ranked according to their antimicrobial activity (*x*-axis; from left to right starting from the most growth inhibitory) and then a running enrichment score for each amino acid residue at each of the four substitution sites is calculated (*y*-axis). Increasing *y*-values indicate the presence of that particular amino acid residue in the ranking segment while decreasing *y*-values indicate the absence. In all cases, the AUC is calculated, whereby positive AUC values represent an enrichment among more active peptides (left side of the *x*-axis) and negative AUC values represent an enrichment among less active peptides (right side of the *x*-axis). An example is shown for the glutamate (E) at position 5 (AUC_E5_). **b** AUC values for each amino acid residue substitution. Effects on antimicrobial activity are binned empirically: very positive (yellow; AUC ≥ 0.2), positive (green; AUC ≥ 0.07), no effect (white; −0.07 < AUC < 0.07 = interquartile range of all values), negative (blue; AUC ≤ −0.07), or very negative (purple; AUC ≤ −0.2). Black dots correspond to the Bac7 parental amino acid residue at each position

This means that interpreting the effect of an amino acid residue should not be inferred from a single peptide (one data point in the ranking) but rather using groups of peptides all having the same amino acid residue at a specific position (multiple data points in the ranking). However, amino acid residue substitutions were unevenly introduced in the library; for example, only one peptide incorporated a tryptophan at position 17 or methionine at position 11, while 2685 different peptides incorporated the substitution to glycine at position 1 (Additional file 1: Fig. S4). Thus, to robustly measure the effect of a particular amino acid residue on antimicrobial activity, we investigated whether specific amino acid residues were significantly enriched in peptides with a higher or lower antimicrobial activity using a permutation scheme (Fig. 1b; enrichment calculation is explained in more depth for Fig. 2a, or fully in the “Methods” section). Based on these results, we could not assign a contribution to antimicrobial activity for 250 out of 483 possible single amino acid residues (23 positions x 20 amino acids plus the stop codon leading to truncated peptides; white boxes in Fig. 1b). Out of these 250, we could not draw any statistical conclusion for 100 amino acid residues because they were too underrepresented in the library (Additional file 1: Fig. S4, and Additional file 1: Fig. S5; *p*-value > 0.1, Benjamini-Hochberg adjusted). The remaining 233 amino acid residues showed an influence on antimicrobial activity. Peptides bearing the amino acid residues of the wild-type Bac7 ‘RLPRPR’ sequence at position 9-14 and an arginine residue at position 6 were considerably enriched in the fraction of the highly antimicrobial variants thereby indicating the crucial importance of these amino acids for the activity of Bac7_1-23_ (yellow boxes in Fig. 1b). Additionally, we found that the random incorporation of stop codons at any of the first 16 positions had mostly negative effects on antimicrobial activity, while insertions downstream of position 16 had no negative effect (Fig. 1b). These results point towards a minimal requirement for Bac7-truncates of 16 amino acids by length, previously also reported by others [7]. Interestingly, substituting the wild-type amino acid residues at positions 3, 5, 7, and 15 and at positions 17-23 allowed to considerably increase the antimicrobial activity of the respective peptide variant. Hence, those 11 positions are potential targets for further activity optimization in Bac7_1-23_ (Fig. 1b). However, proposing a Bac7_1-23_ lead compound is hardly possible based on the sequence-activity relationship data obtained from the epPCR library mainly for two reasons: firstly, many amino acid substitutions were too underrepresented in the library to infer statistical significance concerning their effect on antimicrobial activity (Additional file 1: Fig. S4 and Additional file 1: Fig. S5). Secondly, Bac7_1-23_ is already very active, and single, or even double substitution, may not suffice to considerably boost antimicrobial activity in minimal inhibitory concentration (MIC) assays [9]. As only a small portion of the sequence space was covered for simultaneous substitution (combinations), we decided to create in-depth knowledge on amino acid residue combinations in the course of a second DMS round using a focused Bac7_1-23_ library.

### Deep mutational scanning of Bac7_1-23_ using site-directed mutagenesis

To study the focused library, we first performed combinatorial saturation mutagenesis at four positions of Bac7_1-23_ (20^4^ = 160,000 possible variants). From the 11 positions that seemed to offer room for the accommodation of potentially activity-enhancing amino acid residues (Fig. 1b), we chose to saturate positions 5 and 18-20. Position 5 was selected as it is not part of the crucial N-terminal ‘RRIR’ motif important for cellular uptake and ribosomal binding, nor of a conserved core region among proline-rich AMPs [23]. As little is known from crystallization studies about potential interactions of residues downstream of position 16 with the ribosome [23, 24], and we sought to increase knowledge about the effect those C-terminal amino acid residue substitutions, positions 18-20 were also selected.

The focused library was generated by site-saturation mutagenesis, using one NNK codon for position 18 and a mixture of codons NDT, VMA, ATG, and TGG as described by Tang et al. [25] for the remaining positions. This reduced the need for oversampling drastically, as it limits the bias in amino acid distribution compared to less restrictive schemes (e.g., NNK) [26]. We again grew *E. coli* TOP10 cells expressing the entire library in a single flask (*n*=3; Additional file 1: Fig. S6), sequenced the peptide-encoding DNA at the time of induction and 4.5 h later (Additional file 4), and used its relative abundance as a proxy for growth inhibition (comparable to the DMS workflow display in Fig. 1a; Additional file 5). In total, 156,779 Bac7_1-23_ derivatives were traced back thereby indicating 98% coverage.

Analogous to the epPCR library, we then quantified the effect of each amino acid residue substitution on antimicrobial activity. As each amino acid substitution (e.g., alanine in position 5) appeared in roughly 8000 different peptides (1 × 20^3^=8000; one residue in a specific position fixed and combined with the entire set of possible substitutions at the remaining three positions), no permutation scheme had to be used to calculate the respective effect (*z*) on antimicrobial activity. Instead, we directly inferred the effect of single amino acid residue substitutions by a method inspired by gene set enrichment analysis (GSEA) [27]. First, we ranked all peptides according to their antimicrobial activity (log2-fold change; similarly to Fig. 1a) and then drew enrichment curves for each amino acid residue (Fig. 2a). From those curves, the area under the curve (AUC) was computed giving values between −1 and 1. Positive AUC values indicate enrichment of a specific amino acid residue in peptides with higher antimicrobial activity (left side of the *x*-axis in Fig. 2a), while negative AUC values indicate enrichment in peptides with lower antimicrobial activity (right side of the *x*-axis in Fig. 2a). Amino acid residues with AUC values between −0.07 and 0.07 (covering the interquartile range (IQR), or middle 50%, of all AUC values) did not affect the enrichment and thus on antimicrobial activity.

Our results indicated that peptides that had alanine, glycine, or arginine residues at any of the four positions experienced negative or very negative effects on antimicrobial activity, while isoleucine and tyrosine had positive or very positive effects (Fig. 2b). However, even though these analyses revealed the effect of each single amino acid residue substitution at all four positions in higher detail, they did not reveal the effect of residue combinations, likely to be substantial for generation if an antimicrobial lead compound.

### Effects of amino acid residue combinations

When substituting multiple amino acid residues in proteins or peptides, the effect on function usually equals the sum of the effect of the single substitutions (=additive effects) [28]. In this case, we could select the most activity-enhancing amino acid residues of each position and combine them in one peptide. However, it has been shown that the effect of such combinations can be non-additive [28, 29], that is, it can become larger (cooperative) or smaller (antagonistic) than the sum of the single substitution. Naturally, especially antagonistic combinations have to be avoided.

**Fig. 3:**
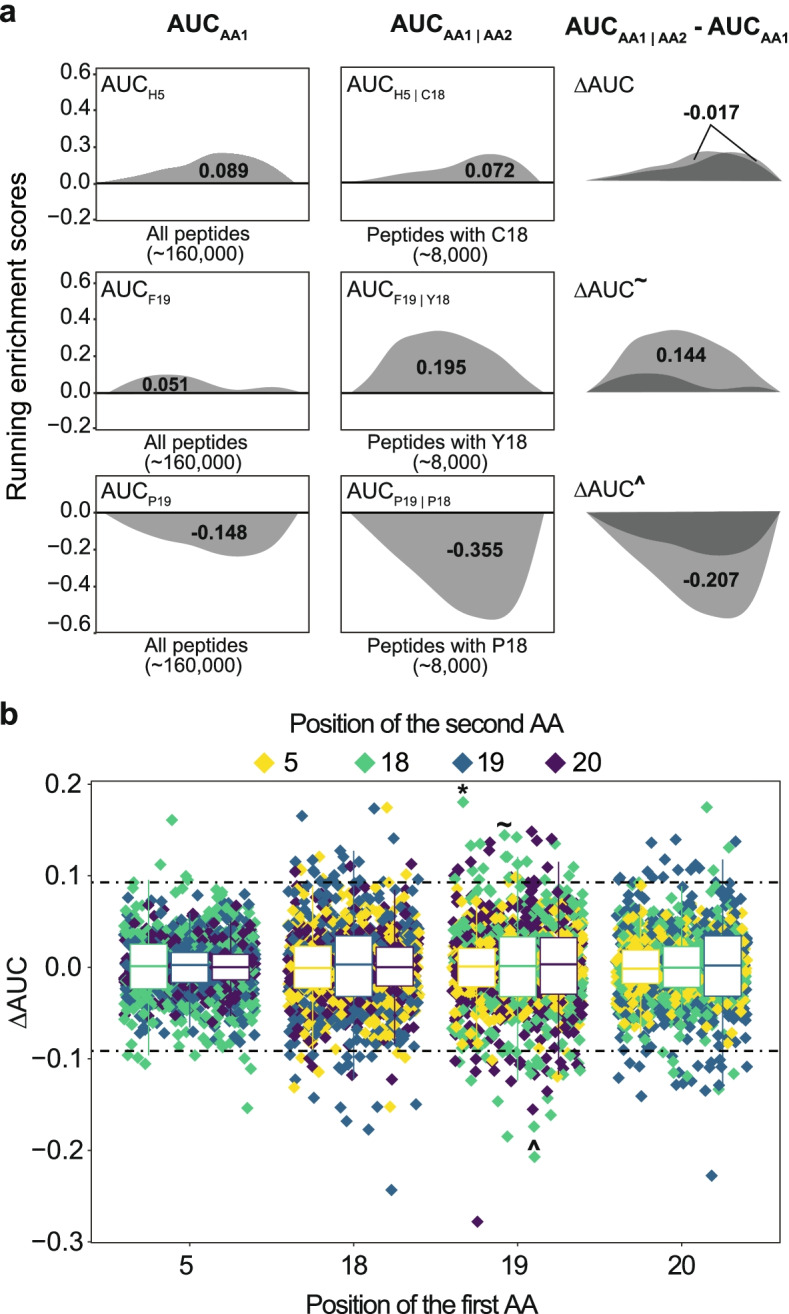
Effect of amino acid residue double combinations. **a** Examples of double amino acid residue combinations resulting in small (top), large positive (middle), and large negative (bottom) ∆AUC values. Left: to calculate the AUC of a single amino acid residue substitution (AUC_AA1_), all peptides are ranked on the horizontal axis according to their antimicrobial activity (as shown in Fig. 2a). Middle: the AUC of the same residue substitution is recalculated for the subset of peptides with a fixed second AA residue at another position (AUC_AA1 | AA2_). Right: the influence of the second amino acid on the effect on antimicrobial activity of the first amino acid is calculated by subtracting the two previous calculations, resulting in ∆AUC. Indicators ~ and ^ are used to link the values to Fig. 3b. **b** ∆AUC values for all 4800 amino acid residue combinations. For each of the 20 amino acid residues at each of the four positions, there are 60 (20 amino acid residues at the remaining three positions) combinations with a second amino acid residue. Non-additive effects are estimated if a second amino acid residue changes the effect that a first amino acid residue has on antimicrobial activity, resulting in either larger positive or negative ∆AUC values. Cooperativity is measured when ∆AUC is larger than 0.09. Antagonism is measured when ∆AUC is smaller than −0.09 (=outliers of a boxplot containing all results; IQR ± 1.5 *IQR). Exemplary ∆AUC values: *P19 and C18, ~F19 and Y18, ^P19 and P18

To investigate if such phenomena occurred, we measured if the effect on antimicrobial activity of one amino acid residue substitution (AUC_AA1_; as shown in Fig. 2a, b) changed upon conditioning the same calculation on a second substitution at another position (AUC_AA1 | AA2_). This change can be described as ∆AUC_AA1&AA2_=AUC_AA1 | AA2_–AUC_AA1_. For example, we compared the effect on antimicrobial activity for the single substitution histidine at position 5 (AUC_H5_ = 0.089; Fig. 3a) among all peptides (~160,000) to the effect on antimicrobial activity of histidine at position 5 among peptides (~8000) that have a cysteine residue at position 18 (AUC_H5| C18_ = 0.072; Fig. 3a). Note that the latter set of peptides is a subset of the former. In this case, ∆AUC_H5&C18_ is small (−0.016), suggesting the effect of histidine in a peptide does not change if cysteine is positioned at position 18, i.e., the combination behaved additively (Fig. 3a). Comparing the ∆AUC of all double combinations (4800) indicated that 96.1% (4613) behaved additively, (−0.09<∆AUC<+0.09 = within IQR ± 1.5 × IQR; Fig. 3b; all data in Additional file 6). The remaining 187 combinations displayed non-additivity, of which 83 combinations showed cooperativity (∆AUC>+0.09), and 104 combinations showed antagonism (∆AUC<−0.09). As exemplarily illustrated (Fig. 3a), we discovered cooperativity for phenylalanine on position 19 and tyrosine at position 18 (∆AUC_F19&Y18_ =+0.144) and antagonism for two prolines at position 18 and 19 (∆AUC_P19 &P18_ =−0.207). In fact, two prolines at any of the positions 18, 19, and 20 behaved antagonistically (Additional file 6). Single substitutions to tyrosine had a positive or very positive effect on antimicrobial activity (Fig. 2a), but combining two tyrosines, e.g., at positions 18 and 19 (∆AUC_Y18&Y19_ = −0.099), 5 and 18 (∆AUC _Y5&Y18_ =−0.152), or 18 and 20 (∆AUC _Y18&Y20_=−0.133), was antagonistic. In general, we observed most non-additive effects among aromatic amino acid residues (57 times), proline (24 times), and arginine (18 times), and very few non-additive effects among serine (0 times), valine (1 time), and glutamate (2 times) (Additional file 1: Fig. S7). Moreover, non-additivity occurred more frequently for neighboring amino acids, e.g., at positions 19 and 20, or 18 and 19 (see boxplots in Fig. 3b). Interestingly, we also discovered cooperativity between the proline and phenylalanine residues (∆AUC _P19&F20_=0.140) at positions 19 and 20 both being part of the Bac7_1-23_ wild type sequence. We hence showed that for design of optimized peptide variants, single substitutions cannot always be combined as antagonistic effects could strongly limit the antimicrobial activity of the peptide.

### Design of the optimized variant Bac7PS

Cooperativity alone (∆AUC values>0.09) cannot be used for the design of highly optimized peptide variants because these effects also appeared among peptide variants with low activity. For example, even though the strongest cooperativity resulted from the combination of proline at position 19 with cysteine at position 18 (∆AUC _P19&C18_ = + 0.18; asterisk in Fig. 3b), proline at position 19 occurred mostly among less active peptides (AUC_P19_ = −0.149; Fig. 2a, b).

We thus aimed to extract a design for the most optimized peptide variant using significant pattern mining [30]. This method looks for significantly enriched combinations of three amino acid residues among the 10% and 25% (arbitrary threshold set by us; Table 1; full dataset in Additional file 7) most and least antimicrobial peptides. A specific combination of four amino acid residues cannot be enriched in a fraction, as it appears only in a single peptide. In addition, as also previously stated, single data points in an NGS-based ranking can be error-prone. We thus investigated enrichment among all triple amino acid residue combinations (in total 32,000 = ${20}^3\times \left(\genfrac{}{}{0pt}{}{4}{3}\right)$) shown in Additional file 7), each appearing in 20 different peptides (20 possible combinations for the fourth amino acid residue). Peptides with the triple combination of alanine-proline-proline residues at positions 5, 18, and 19 were the least active peptides in the entire library (Table 1). Indeed, our previous analysis showed that combining two prolines at positions 18 and 19 was strongly antagonistic for activity (∆AUC_P19 &P18_ =−0.207; Fig. 3a, b). Additionally, we found that, even though single tyrosine residues were the most positive substitutions for antimicrobial activity (Fig. 2a, b), peptides with combinations of three tyrosine residues were not among the most active peptides (two examples shown in Table 1). This can be explained by the measured antagonism in combinations of two tyrosine residues shown before. We recorded the highest antimicrobial activity among peptides bearing tyrosine-phenylalanine-methionine at positions 18–20, including cooperativity (∆AUC_F19&Y18_ =+0.144; Fig. 3a, b), or asparagine-histidine-asparagine at positions 18–20, including cooperativity (∆AUC_H19&N18_ =+0.108) (Table 1).

**Table 1:** Significant pattern mining results

Rank	Position 5	Position 18		Position 19	Position 20	# in 25% ***most*** actives	# in 25% ***least*** actives	# in 10% ***most*** actives	# in 10% ***least*** actives
1	-	Y		F	M	20	0	20	0
2	-	N		H	N	20	0	20	0
3	C	N		N	-	20	0	19	0
1602	Y	Y		-	Y	11	0	7	0
4815	Y	Y		Y	-	10	0	3	0
32,000	A	P		P	-	0	20	0	20

A subset of triple amino acid combinations was obtained by significant pattern mining (see Additional file 7 for all combinations). The first column indicates the rank of each combination (from most to least significantly enriched in 10% most inhibitory peptides by *p*-values). Columns 2–5 indicate the amino acid residue at each position in the triple combination. “–” indicates the open fourth position of the peptide. Columns 6–9 indicate the number of peptides containing the respective combination among the top 25% and top 10% most and least active peptides

To design an optimized peptide variant as our lead compound, we build a peptide containing asparagine-histidine-asparagine at positions 18–20. This was the most significantly enriched triple combination among highly activity peptides in the library and incorporated one of the largest cooperative effects. For the remaining position 5, we avoided a second tyrosine and chose isoleucine because it showed the second most positive effects on growth inhibition at position 5 (Fig. 2a, b). In addition, among all 20 variants containing the chosen triple combination in the library, the peptide containing isoleucine at position 5 was the most active (Additional file 5). This Bac7_1-23_ P5I R18Y L19F P20M variant is from here on referred to as Bac7PS.

### Characterization of Bac7PS and Bac7_1-23_

**Fig. 4:**
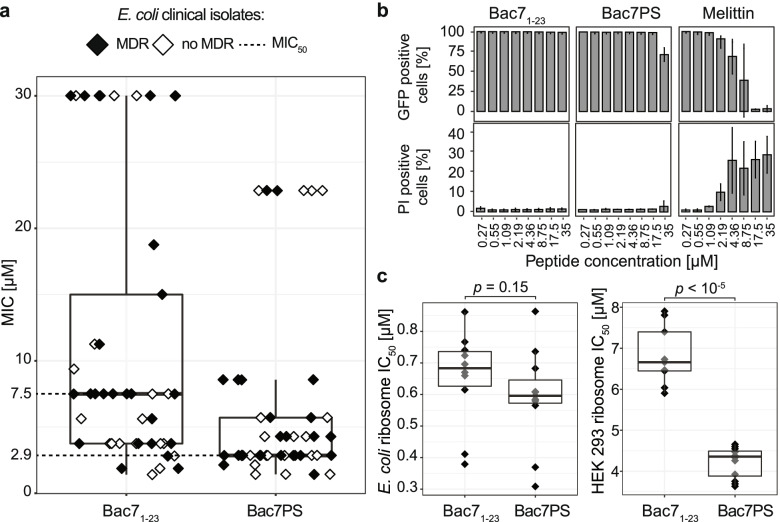
Characterization of Bac7PS and Bac7_1-23_. **a** MICs of a panel of clinical isolates of *E. coli* (*n*=45) including MDR bacteria (ESBL, CRE, *n* = 25). MIC_50_ of Bac7_1-23_ = 7.5 μM, MIC_50_ of Bac7PS = 2.9 μM. **b** Membrane damage assays measuring GFP loss (% of cells that lost GFP fluorescence) and PI uptake (% cells that gained PI fluorescence) when incubating *E. coli* TOP10 in MHB II for 30 min in the presence of increasing concentrations of Bac7PS and Bac7_1-__23_ (*n* = 3; error bars = 1SD). MIC of melittin against *E. coli* TOP10 is 5.0 μM (data not shown). **c** In vitro translation inhibition assays against *E. coli* ATCC 29522 (left) and HEK 293 ribosomes (right). IC_50_ values are extracted from a luminescence assay translating firefly luciferase mRNA peptide concentrations between 800 and 0.08 μM (*n* = 9). *p*-values (*p*) are calculated by performing a Wilcoxon rank-sum test, testing for differences in mean IC_50_ values of Bac7_1-23_ and Bac7PS

To investigate if our optimization strategy resulted in a potential antimicrobial drug lead compound with improved activity over Bac7_1-23_, we chemically synthesized Bac7PS (92% purity) and Bac7_1-23_ (95% purity) and characterized them further. While strictly adhering to CLSI standards [31], we first evaluated the antimicrobial activity of the peptides against the microbial pathogen model used for DMS (*E. coli* TOP10) in MIC assays. The set was completed by *E. coli* ATCC 25922, a quality control strain often used in clinical microbiology, the transporter-loss mutant *E. coli* BW25113 Δ*sbmA* [32], which is less susceptible to Bac7_1-23_ [33], and its parental strain *E. coli* BW25113 [32]*.* Remarkably, we found an approximately twofold reduction of the MIC with Bac7PS for the DMS strain *E. coli* TOP10 (MIC of 2.1 μM for Bac7PS, 4.6 μM for Bac7_1-23_). Furthermore, Bac7PS showed a MIC of 14.4 μM against the transporter-loss mutant BW25113 Δs*bmA*, while Bac7_1-23_ was only active at the highest tested concentration of 52.1 μM (Table 2). Interestingly, the MIC for the quality control strain was similar for both peptides (2.6 μM for Bac7PS, 2.8 μM for Bac7_1-23_). Next, we recorded the MICs of both peptides against a panel of 45 *E. coli* clinical isolates also containing 23 MDR strains expressing extended-spectrum beta-lactamases (ESBL) or carbapenemase (CRE) collected from Swiss hospitals. The results indicated that the antimicrobial activity of Bac7PS exceeded that of Bac7_1-23_ with an MIC_50_ for Bac7PS of 2.9 μM as compared to 7.5 μM for Bac7_1-23_ (Fig. 4a), hence proofing the superiority of the DMS optimized variant Bac7PS.

**Table 2:** Summary of susceptibility assays

	MIC against ***E. coli*** strains [μM]					Hemolysis of mouse red blood cells [%]		Toxicity IC_**50**_ [μM]		TI
Peptide	TOP10	ATCC 25922	BW 25113	BW 25113 ∆***sbmA***	Clinical isolates (MIC_**50**_)	1xMIC	4xMIC	HeLa	HEK 293	Toxicity/MIC
Bac7_1-23_	4.6	2.8	7.4	52.1	7.5	2.1%	6.4%	1460	1970	195
Bac7PS	2.1	2.6	3.6	14.4	2.9	3.1%	3.8%	521	755	180

MIC values are averaged (*n*>3) and performed under CSLI standards in the MHB II medium. Hemolysis assays and toxicity measurements were performed in triplicates. The therapeutic index (TI) is calculated by dividing the IC_50_ values measured with HeLa cells by the MIC_50_ obtained from clinical isolates (see Fig. 4a)

We next investigated whether an increased propensity to damage membranes could be the reason for the improved antimicrobial activity of Bac7PS. Membrane damage is the most frequent way AMPs kill bacteria and is often considered the reason for high toxicity against eukaryotic cells [4]. However, Bac7 is known to not damage bacterial membranes at its MIC [12]. Consequently, we investigated if Bac7PS had acquired a tendency to damage membranes, which could imply a greater risk for systemic human applications. Cell damage was assessed by measuring leakage of green fluorescent protein (GFP), expressed in *E. coli* TOP10 cells, and uptake of (membrane-impermeable) propidium iodide (PI) [34]. For both peptides, we confirmed that the integrity of the membrane is neither affected at the MIC nor 8-fold higher concentrations (~1% PI-positive cells for Bac7_1-23_ and Bac7PS, ~99% of cells retained GFP levels; Fig. 4b). In contrast, the known membrane-active peptide melittin rapidly induced PI uptake and loss of GFP at its MIC (Fig. 4b). However, at approximately 16-fold above its MIC (~35 μM), we noticed minor membrane damage of cells only treated with Bac7PS, indicated by loss of GFP (26% of cells lost GFP) and minor uptake of PI (~3% PI-positive cells). This effect was more pronounced when comparing these peptides in MHB I medium, which is not cation-adjusted and therefore less ionic, an effect that is often taken advantage of to increase membrane interaction of cationic AMPs [35, 36] (Additional file 1: Fig. S8). In conclusion, the growth inhibitory activity of Bac7PS was not due to membrane damage.

To ensure that mammalian membranes were also not affected, we performed a hemolysis assay using red blood cells from mice. The results indicated that only a low fraction of membranes lysed (<4%) when applying Bac7PS and Bac7_1-23_ at the MIC measured for *E. coli* ATCC 25922, and only very minor lysis (<7%) when exceeding the MIC fourfold (Table 2). The reduced dependency on the inner membrane transporter SbmA (Table 2) and the slight increase of membrane damage at very high concentrations (Fig. 4b and Additional file 1: Fig. S8) indicated a higher degree of membrane interaction of Bac7PS compared to Bac7_1-23_. This could potentially lead to facilitated uptake of Bac7PS and thus a decreased MIC of the peptide.

While low MIC values are desirable for an antimicrobial compound, low toxicity to eukaryotic cells is imperative. To measure the general toxicity of both peptides, we added them to human epithelioid cervix carcinoma (Henrietta Lacks = HeLa) and human embryonic kidney 293 (HEK 293) cells and quantified the degree of reduction of 3-(4,5-dimethylthiazol-2-yl)-2,5-diphenyltetrazolium bromide (MTT), only catalyzed by metabolically active and viable cells [37]. Bac7PS showed an increase in toxicity against HeLa with an IC_50_ of 521 μM compared to an IC_50_ of 1460 μM for Bac7_1-23_ (Table 2). Toxicity values against HEK 293 cells were similar when compared to HeLa cells, with an IC_50_ of 755 μM for Bac7PS compared to an IC_50_ of 1970 μM for Bac7_1-23_ (Table 2). However, the TI (Table 2) remained very high (>180) for both peptides, especially when compared to most other AMPs, suggesting low toxicity at therapeutically valuable concentrations [38].

Furthermore, we investigated the effect of the amino acid substitutions in Bac7PS on the expected main activity, bacterial protein synthesis inhibition. For this, Bac7PS and Bac7_1-23_ were incubated with bacterial S30 extracts (*E. coli* ATCC 25922) together with a luciferase encoding mRNA. To determine target selectivity for the bacterial ribosome, we also performed the same experiment using S30 extracts from HEK 293 cells. We measured the resulting luminescence of each sample (peptide concentration range: 800 to 0.08 μM in 2.5-fold dilutions steps; *n* = 9) and extracted the IC_50_ value, the concentration at which half-maximal protein translation inhibition was achieved. Bac7PS triggered a mean 10% increase of the activity of *E. coli* ribosomes (Fig. 4c) compared to Bac7_1-23_, which, however, did not reach statistical significance (*p*-value = 0.15, Wilcoxon rank-sum test). For HEK 293 ribosomes, Bac7PS showed a mean 38% increase in ribosomal binding (Fig. 4c) compared to Bac7_1-23_ (*p*<10^−5^, Wilcoxon rank-sum test), but at much higher concentrations compared to the bacterial ribosome. However, in a HEK 293 whole-cell assay, increased translational inhibition of Bac7PS relative to Bac7_1-23_ only appeared at concentrations above 200 μM (Additional file 1: Fig. S9). At this concentration, we also observed general toxicity against HEK 293 cells (IC_50_ values in Table 2), which suggests that cell viability was rather affected by other mechanisms than translational inhibition (e.g., membrane damage). Thus, Bac7PS remained a very strong, but still selective ribosomal inhibitor for bacteria.

### Bac7PS activity in a murine model

**Fig. 5:**
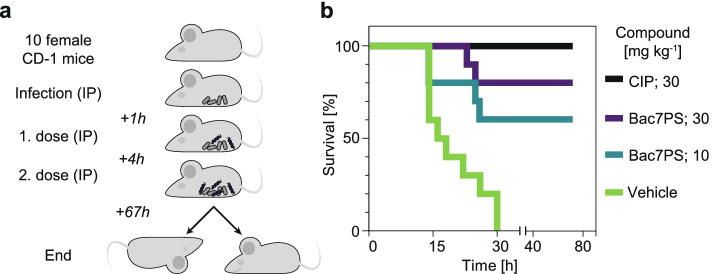
Efficacy of Bac7PS in a murine model. **a** Efficacy study scheme. Drugs are applied to mice infected with *E. coli* ATCC 25922. Bacteria and drugs are administered intraperitoneally (IP). **b** Survival rates after infection. Mice are infected with *E. coli* ATCC 25922 and then treated with CIP as positive control and without a drug (vehicle) as the negative control. The study was performed for each condition as described in **a**

Finally, we investigated the efficacy of Bac7PS in a murine septicemia infection model (Fig. 5a). The maximal dose at which all CD-1 mice survived intraperitoneal Bac7PS treatment for 3 days was 50 mg kg^−1^ (Additional file 1: Fig. S10). Additionally, we saw that a second administration of 40 mg kg^−1^ Bac7PS was also tolerated by the animals (data not shown). For the efficacy study (Fig. 5a), we used two concentrations of Bac7PS and applied them twice 4 h apart: 30 mg kg^−1^, a concentration close to the maximum tolerated dose, and 10 mg kg^−1^. IP infection of CD-1 mice using *E. coli* ATCC 25922 resulted in the death of all 10 mice after 72 h if treated using the vehicle control and survival of all mice when treated with 30 mg kg^−1^ ciprofloxacin (CIP) (Fig. 5b). Bac7PS showed a dose-dependent efficacy with 80% and 60% survival at administrated concentrations of 30 and 10 mg kg^−1^, respectively.

Taken together, our optimization strategy guided us in the design of an antimicrobial lead compound with activity improvements over Bac7_1-23_, which has to be further evaluated when entering additional drug development stages.

## Discussion

DMS methods have been widely applied to study the sequence-activity relationship of proteins [39], optimize enzymes [40], or design therapeutics [41]. One of the major potential advantages of implementing DMS for the development of AMPs to antimicrobial drug candidates is that a large number of peptide variants can be functionally assessed without the need for their production and purification using chemical or biological methods. This advantage can be exploited to largely increase the sequence space assessed for studying sequence-activity relationships eventually allowing hit-to-lead optimization of AMPs.

About the applied intracellular screening method, we note that intracellular bacterial self-screenings might lead to identifying better binders to intracellular targets, variants with increased membrane damaging properties, or variants that reach higher intracellular concentration because of increased solubility, mRNA stability, or proteolytic resistance. However, uptake across the outer and inner membrane, for example via the SbmA transporter, cannot be selected for. As for the proline-rich peptides used in this study, the N-terminal “RRIR” motif seems to be most critical for cellular uptake [8, 42], we decided not to modify this part and accepted the risk for contracting unknown effects regarding uptake when modifying other regions of Bac7_1-23_. Still, even if intracellularly produced peptides would later fail to pass over the bacterial membrane, uptake could perhaps be recovered post-screen, for example by fusing them to motifs of cell-penetrating peptides [43].

We present an in-depth characterization of the importance of the antimicrobial activity of each amino acid residue substitution in Bac7_1-23_. Compared to generating each amino acid residue substitution in a peptide individually, we used an epPCR for library generation, which also resulted in a large number of highly mutated (e.g., 38% were quadrupole mutants; Additional file 1: Fig. S3) Bac7_1-23_ variants. Measuring the antimicrobial activity of heavily mutated variants was valuable to determine the sequence-activity relationship of Bac7_1-23_ because (i) the activity of specific amino acid residue substitution can be robustly determined by averaging the activity of thousands of variants sharing the same specific exchange, and (ii) it allows to obtain information on the possibility to incorporate additional mutations in the same peptide. Much of the information acquired upon parallelized intracellular expression of Bac7_1-23_ variants is in high agreement with previously reported data (Fig. 1b). Except position seven, we retrieved the core motif of Bac7_1-23_ between positions 9 and 14, known to be largely evolutionarily conserved among proline-rich AMPs [5]. By the random insertion of stop codons, we found that peptides shorter than 16 amino acids are mostly inactive. As the peptides in our assay are synthesized in the cytosol, we attributed the loss of activity to weaker inhibition of protein translation, which is supported by earlier research [42], but contested in recently made claims attributing the loss of antimicrobial activity of Bac7_1-15_ to impaired uptake into the cytosol [5, 7]. Strikingly, we found that the C-terminus of Bac7_1-23_ offered a large potential for optimization even though most research currently focuses on the substitution of the first 16 residues [9, 10]. Unlike the core motifs of proline-rich AMPs, which well overlapped in the crystal structure when bound to the ribosome, the residues of the C-termini of proline-rich AMPs are less conserved [23]. The higher spatial flexibility of the upper ribosome chamber is illustrated by the fact that also Gram-positive targeting macrolide antibiotics bind in that region [44] and that longer Bac7 variants (60 or 35 amino acids by length) showed a broader (against different Gram-negative bacteria) antimicrobial activity than their shorter counterparts [8]. Surprisingly, we found that the insertion of a tyrosine residue is one of the most activity-enhancing single amino acid residue substitutions in the site-specific library, especially at positions 18 and 19 (Fig. 2b). Interestingly, even though a tyrosine residue is not present in full-length Bac7 comprised of 60 amino acids, it is common among other proline-rich AMPs [5, 45], potentially because, similarly to arginine residues, an aromatic residue can well fill the space and interact with the amino acid residues or ribonucleotides of the surrounding ribosomal exit tunnel [9].

Our quest towards the identification of non-additive effects in intracellular activity screenings allowed the design of Bac7PS bearing two amino acids that cooperatively contributed to antimicrobial activity. While not researched well in the peptide field, results from directed evolution of proteins indicate that non-additive (i.e., epistatic) effects, must be considered to fully optimize a given scaffold for a specific function [46]. However, other than proteins, which usually adopt complex structural confirmation, linear peptides display less structural flexibility, potentially leading to a reduced propensity for interactions between amino acid residues. Still, our results prove that non-additivity occurs (Fig. 3a/b). We reckon that it might predominately result from phenomena such as the modulation of the affinity for molecular targets (e.g., macromolecules or membranes), components of the cellular defense machinery (e.g., drug efflux pumps or proteases), or exceedance of crucial physicochemical parameters (e.g., solubility). We hence hypothesize that also in the peptide field, non-additive effects are of considerable importance.

Bac7PS excelled over Bac7_1-23_ in terms of antimicrobial activity against clinical isolates including MDR bacteria and a decreased dependency on the SbmA transporter (Fig. 4a; Table 2). As Bac7_1-23_ was largely inactive against the SbmA transporter knockout strain, this improvement is a very favorable new property, potentially making bacterial resistance development harder. We mainly attribute the activity gain to the increased ability to penetrate membranes, which was accompanied by a slight increase in membrane damage at higher concentrations compared to Bac7_1-23_ (Fig. 4b; Additional file 1: Fig. S8; Table 2). This effect likely correlates with the observed cooperativity between the phenylalanine and tyrosine residues. Our hypothesis could be supported by the fact that aromatic amino acids, such as the now introduced tyrosine residue at position 18 or the phenylalanine residue at position 19, are known to enhance membrane penetration [47]. Similar beneficial effects were observed in the study of Mardirossian et al., relying on the activity assessment of 133 chemically synthesized Bac7_1-16_ (16 amino acids long) derivatives [9]. The most promising candidate mentioned in that study, a peptide called B7-005 incorporating multiple tryptophan residues, was also more active against a broader panel of bacteria including a SbmA knockout. However, B7-005 was not more active than Bac7_1-16_ against the initial screening strain and seemed to show slightly decreased inhibition of protein translation. On the contrary, Bac7PS showed increased activity against the screening strain *E. coli* TOP10 and an at least equally strong inhibition of protein translation than the wild-type peptide Bac7_1-23_. Bac7PS displayed a small (non-significant) shift in binding the *E. coli* ribosome (+10%; *p* = 0.15) while binding to the human ribosome was more clearly improved (+38%; *p* < 10^−5^) (Fig. 4c). However, as toxicity and translational inhibition remained low for human cells (Table 2; Additional file 1: Fig. S9), there appears to be a large window at which bacteria can be efficiently eradicated with no detectable impairment of human cell viability. We assume that the absence of the SbmA transporter in human cells, as well as structural differences of the bacterial and mammalian membrane and cell surface, is responsible for the observed insensitivity of human cells, corroborating the potential for Bac7PS as an antimicrobial candidate.

Lastly, it is hard to judge if the activity increase of Bac7PS compared to Bac7_1-23_ measured in vitro can be translated to in vivo experiments. There has been one report examining the efficacy of Bac7_1-35_ (35 amino acid long) in vivo, which, however, does not allow for direct comparison to our results [48]. The authors treated mice, infected with Gram-negative *Salmonella typhimurium* intraperitoneally, with 30 mg kg^−1^ of Bac7_1-35_ and showed an efficacy of 36%, and increased the mean survival from 10 days (untreated control) to 24.5 days (treated). In our study, all untreated mice already died within 30 h after an intraperitoneal injection of *E. coli*. However, we demonstrated a treatment efficacy of 60% when applying 10 mg kg^−1^ and 80% when applying 30 mg kg^−1^ of Bac7PS. More studies, especially on the pharmacokinetics and using different infection models are needed to evaluate the suitability of Bac7 variants and Bac7PS for further antimicrobial drug development stages.

## Conclusions

In summary, we provided an example of a successful AMP hit-to-lead optimization by DMS, resulting in an antimicrobial lead compound. Bac7PS is a strong ribosomal inhibitor that is non-toxic to human cells and displays a far higher TI than most other AMPs. Compared to Bac7_1-23_, which already displays high activity against pathogenic bacteria, Bac7PS has increased activity against multidrug-resistant clinical isolates. The generated sequence-activity relationship landscape might inspire further engineering approaches on Bac7_1-23_, and the principal strategy could be easily expanded for optimization of other intracellularly active, or even membrane damaging AMPs if displaying the peptides on the surface [19]. We envision coupling this platform to target-based discovery, to directed evolution approaches, or to further increase the activity spectrum of AMPs when directly expressing the peptides in drug-resistant strains.

## Methods

### Chemicals and reagents

Unless otherwise stated, all chemicals, reagents, and primers were obtained from Sigma Aldrich (Buchs, CH). Restriction enzymes and their buffers were obtained from New England Biolabs (Ipswich, USA). Kits for the isolation of plasmid isolation and DNA purification kits were obtained from Zymo Research (Irvine, USA). Peptides in either purified (>90%) or crude form were obtained from Pepscan (Lelystad, NL) or Genescript (Piscataway Township, USA). Sanger sequencing was done at Microsynth (Balgach, CH).

### Bacterial strains and cultivations

Unless otherwise stated, all experiments were performed using *E. coli* TOP10 (F^–^ *mcr*A Δ (*mrr*-*hsd*RMS-*mcr*BC) φ80*lac*ZΔM15 Δ*lac*X74 *rec*A1 *ara*D139 Δ(*ara-leu*)7697 *gal*U *gal*K λ^–^ *rps*L(Str^R^) *end*A1 *nup*G; Thermo Fisher Scientific, Waltham, USA). In this study, overnight cultivations were performed either in 14-ml polypropylene tubes (Greiner, Kremsmuenster, AT), filled with 5 ml of lysogeny broth (LB) medium (Difco, Becton Dickinson, Franklin Lakes, USA) or in 96-deep-well polypropylene plates (Greiner, Kremsmuenster, AT) filled with 500 μl of LB medium. All samples were incubated at 37°C with agitation on a shaker (Kuhner, Birsfelden, CH) operated at 200 r.p.m. and 25 mm amplitude. All media were supplemented with the appropriate antibiotic for plasmid maintenance (50 μg ml^−1^ kanamycin; 100 μg ml^−1^ carbenicillin) and 1% (w/v) d-glucose for repression of gene expression from catabolite-repression sensitive promoter P_BAD_. In the case of peptide expression experiments, cultures were incubated without d-glucose and 0.3% (w/v) of the inducer l-arabinose was used for induction. For all cultivations on solid medium, 15 mg ml^−1^ agar (Difco) was added to the broth, and incubation was performed in an incubator (Kuhner) at 37°C. If not indicated differently, the optical densities (OD) of bacterial cultures were determined by measuring light scattering at 600 nm using a UV/VIS spectrophotometer (Eppendorf, Hamburg, DE).

### Generation of the randomly mutagenized Bac7_1-23_ library

To mutate the Bac7_1-23_ gene randomly, we used epPCR. We amplified the Bac7_1-23_ gene using primer 1 and primer 2 (Additional file 1: Table S1), which bind upstream of the first codon, including the start codon, and downstream of the last codon, not including the stop codon. For amplification, we used the *Pfu* DNA Polymerase exo^−^ mutant (D141A/E143A), *Pfu* reaction buffer including dNTPs, and 0.3 mM final concentration of MnCl_2_ to increase the error rate. The amplification was performed using 30 cycles of 98°C for 10 s, 60°C for 15 s, and 72°C for 10 s. The PCR product (118 bp in length) was purified and used for a restriction digest using enzymes HindIII-HF and PstI-HF. The digested product was again purified using a DNA purification kit and ligated to plasmid pBAD [49] (Thermo Fisher Scientific) previously digested with the same enzymes using T4 ligase (800 units). The ligation product was used to transform 20 μl of CloneCatcher™ Gold DH5G Electrocompetent *E. coli* (Genlantis, Burlington, USA) cells using electroporation. Recovered cells were plated and incubated overnight on LB agar plates supplemented with carbenicillin. Approximately 5.6 million colonies were washed off several plates using LB medium and the plasmids containing the peptide-encoding DNA sequences were extracted from 2.5 × 10^9^ cells. An aliquot of 5 ng of these plasmids was used to transform *E. coli* TOP10 cells. Approximately 10 million colonies were recovered from the plates after overnight incubation by washing with LB medium. The suspension was diluted to OD = 1 with LB medium, glycerol was added to a final concentration of 20% (v/v), and aliquots of 500 million cells were stored at −80°C.

### Generation of the site-saturation Bac7_1-23_ library

A focused strategy was pursued to generate diversity at positions 5, 18, 19, and 20 in the Bac7_1-23_ peptide. These sites were simultaneously randomized on the genetic level by site-saturation mutagenesis using a single NNK codon for position 18 and a mixture of codons as suggested in the small intelligent approach [25] for the remaining three positions (see Additional file 1: Table S1 for sequences and mixing ratios). Degeneration was introduced by using the QuikChange technique [50], amplifying the pBAD plasmid containing the Bac7_1-23_ gene using a mixture of 20 oligonucleotides (Additional file 1: Table S1) at a final concentration of 0.3 μM and Phusion® High-Fidelity PCR Master Mix in HF buffer. The amplification was performed using 20 cycles of 98°C for 10 s, 60°C for 15 s, and 72°C for 10 s. The PCR product was treated with the enzyme Dpn1 to remove the template plasmid and subsequently purified. The purified product was used to transform 20 μl of CloneCatcher™ Gold DH5G Electrocompetent *E. coli* (Genlantis, Burlington, USA) cells using electroporation. Transformation, recovery, and storage were performed as explained for the random mutagenesis protocol, but this time we recovered approximately 2.3 million (*E. coli* Clonecatcher) and approximately 1.9 million (*E. coli* TOP10) colonies.

### Parallel growth experiment

To assess the antimicrobial activity of multiple peptides in parallel when expressed intracellularly in *E. coli* TOP10, previously prepared aliquots containing *E. coli* plasmid libraries created by either random mutagenesis or site-saturation mutagenesis were used. For both approaches, three aliquots containing approximately 500 million cells each of *E. coli* TOP10 harboring peptide-encoding DNA sequences on the pBAD plasmid were thawed and added to three 1-l baffled shake flasks containing 100 ml of LB medium + 100 μg ml^−1^ carbenicillin. The cultures were grown for roughly 7 h at 37°C. When the OD reached approximately 0.2, the cultures were supplemented with l-arabinose to a final concentration of 0.3% (w/v) to induce peptide expression. When analyzing the randomly mutagenized library, cell samples were taken from each biological replicate at the point of induction and 4 h post-induction. When analyzing the library created by site-saturation mutagenesis, cell samples were taken from each biological replicate at the point of induction and 4.5 h post-induction. The plasmids were extracted from all samples using a plasmid isolation kit.

### Single-strain growth experiments

To assess the antimicrobial activity of single peptides when expressed intracellularly in *E. coli* TOP10, a monoclonal strain carrying the pBAD plasmid containing a single peptide gene was picked from solid media, incubated overnight, and inoculated into a fresh LB medium containing 0.3% (w/v) l-arabinose to a final OD of 0.01 into 96-well microtiter plate (Greiner). Growth of strains was recorded by measuring OD in a Tecan Infinite 200 PRO (Tecan, Männedorf, CH) for at least 4 h (37°C, 1.5 mm orbital shaking).

### Next-generation sequencing

Peptide-encoding DNA sequences on plasmids, collected from both experiments (three replicates across the different time points) were sequenced using NGS. To first amplify the peptide-encoding DNA, we added primer 1 and primer 2 (Additional file 1: Table S1), 100 ng of plasmid, and thermocycled (10 cycles of 98°C for 10 s, 60°C for 15 s, and 72°C for 10 s). The amplification product was purified using an agarose gel. Single Index PentAdapters from Pentabase were used to prepare PCR-free libraries with the KAPA HyperPrep Kit (Roche, Basel, CH) according to the manufacturer’s specifications. Libraries were quantified using the qPCR KAPA Library Quantification Kit. Libraries were pooled and sequenced single read with 101 cycles using an Illumina NovaSeq 6000 SP flow cell. Roughly 10% genomic PhiX library was used as a spike-in to increase sequence diversity. Base-calling was done with bcl2fastq v2.20.0.422. The resulting fastq files were processed using the software Geneious Prime 2020 (Biomatters, Auckland, NZ) and an in-house software written in R. For the randomly mutagenized library, we first discarded all sequences that missed the combination of a start codon and 69 bases downstream stop codon. Next, we discarded all sequences that appeared less than five times in at least two replicates. Next, all DNA sequences were translated and the resulting peptide sequences were counted for each replicate and time point. All NGS counts can be seen in Additional file 2. For the site-saturation library, we first discarded all sequences that did not have a start codon and 69 bases downstream stop codon, translated the DNA sequences into peptide sequences, aligned them to our reference table of 160,000 possible peptide variants, and counted them for each replicate and time point. All NGS counts can be seen in Additional file 4.

### Ranking of peptides based on antimicrobial activity

To analyze the NGS read counts of both libraries, we used the standard workflow of DESeq2 [51] (NGS read count normalization, dispersion estimates, and Wald’s test). We calculated the log2-fold changes of the NGS read counts (listed for each peptide in Additional files 2 and 4) between the time of induction and 4.0 h (random mutagenesis) as well as 4.5 h (site-saturation mutagenesis) post-induction. A Bayesian shrinkage estimator was employed to shrink the log2-fold change for each sequence using the R/Bioconductor package ‘apeglm’ [52]. Finally, the shrunken log2-fold change was used as a proxy for the antimicrobial activity of each peptide, as the propagation rate of the peptide-encoding DNA would follow the growth rate of the respective host. The more negative a log2-fold change, the higher the observed antimicrobial effect. The ranked peptide list from the randomly mutagenized library and the focused library are in Additional files 3 and 5, respectively.

### Enrichment curve-derived AUCs to quantify the degreed of growth inhibition

To determine the effect of amino acid residue substitutions on antimicrobial activity, we applied a variation of the GSEA [27]. This adjusted method was based on drawing what we refer to here as enrichment curves (Fig. 2a). In those plots, each value on the *x*-axis represented a peptide ranked by their shrunken log2-fold changes, giving rise to the ranked peptide set *L*. More active peptides are assigned to the left spectrum of the *x*-axis (lowest log2-fold change), while less active peptides are assigned to the right spectrum of the *x*-axis. For each single amino acid residue substitution, e.g., alanine at position 1, we defined *S* to be a set of all peptides that exhibit this substitution. Each *y*-value indicated whether the corresponding peptide pertains to the peptide-set or not. Formally, if a peptide *p*_*i*_ in the ranked list *L* pertains to the peptide set *S*, its value is defined as

1 $${P}_{hit}\left(S,i\right)=\sum_{p_i\in S;j\le i}\frac{1}{\mid S\mid }$$

If peptide *p*_*i*_ is not present in the set *S*, its value will correspond to

2 $${P}_{miss}\left(S,i\right)=\sum_{p_i\notin S;j\le i}\frac{1}{N-\mid S\mid }$$

where *N* corresponds to the total number of peptides in the ranked list.

Subramanian et al*.* developed a so-called enrichment score (ES) that is defined as the maximum deviation of *P*_*hit*_ − *P*_*miss*_ from zero. We proposed a slightly different approach, which we referred to as the AUC. Compared to the ES, the AUC describes the complete dynamic of enrichment curves. We computed the AUCs as follows:

3 $$AUC(S)=\frac{1}{\mid L\mid}\sum_{i=1,\dots, \mid L\mid}\left[{P}_{hit}\left(S,i\right)+{P}_{miss}\left(S,i\right)\right]$$

Positive AUC values indicated that the corresponding set *S* is overrepresented in peptides with higher antimicrobial activity (top of the list), while negative AUC values indicate that the corresponding peptide set is overrepresented in peptides with lower antimicrobial activity (bottom of the list). AUC values close to zero indicated that the peptide set was randomly distributed across the list, or exhibited bimodal behaviors.

In cases where the peptide-set *S* is small, i.e., if there are only a few observations for an amino acid residue substitution, the computation of AUCs can be misleading. To present a more robust measurement for the randomly mutagenized Bac7_1-23_ library, we resorted to a permutation scheme that allowed us to derive better estimates of the effect of each amino acid residue substitution on activity. These permutation schemes relied on drawing from the null distribution, i.e., assuming that a single substitution, in the following denoted as *S*_*M*_, is not overrepresented in peptide with higher or lower antimicrobial activity. The following scheme is executed for each permutation:

- Randomly permute the peptides in list *L*, giving rise to permuted list $\hat{L}$, which destroys the activity-based ranking
- Compute enrichment curves for permuted list $\hat{L}$ and single substitution *S*_*M*_
- Compute the permuted $\hat{AUC}\left({S}_M\right)$

This scheme is repeated *n*_*perm*_ times, where commonly *n*_*perm*_ = 10^3^, or 10^4^, giving rise to the null distribution of the AUC values. *z*-scores were derived as:

4 $${z}_{S_M}=\frac{auc(S)-\mathrm{mean}\left(\hat{auc}\left({S}_M\right)\right)}{\mathrm{std}\left(\hat{auc}\left({S}_M\right)\right)}$$

*z*-scores represented how many standard deviations the observed AUC value differed from the mean of all AUC values derived from the null distribution. A large positive *z*-score indicated an enrichment of *S*_*M*_ among more active peptides, and a large negative *z*-score denotes enrichment of *S*_*M*_ among less active peptides. We furthermore computed a two-sided *p*-value (${p}_{S_M}$) to assess the statistical significance of the observed measurements under the null hypothesis. It is defined as

5 $${p}_{S_M}=\frac{\#\mathrm{abs}\left( auc\left({S}_M\right)\ge \hat{auc}\left({S}_M\right)\right)}{n_{perm}}$$

To account for multiple testing, *p*-values were adjusted using the Benjamini-Hochberg procedure with a false discovery rate of *α* = 0.1.

### Determination of non-additivity

The AUC_AA1_ describes the effect of a single amino acid residue substitutions (AA1) on antimicrobial activity and can be calculated as described in Eqs. (1-3). We defined the conditional AUC_AA1 | AA2_ as the effect of a single amino acid residue substitutions (AA1) on antimicrobial activity using a ranked peptide set *L*′ containing only peptides that have a fixed amino acid residue (AA2) at a specific position. To determine if two amino acid residues behave non-additively, we calculated the difference of the AUC calculated for the conditional ranked peptide set *L*′ (with a size of approximately 8000 peptides) and the AUC calculated for the entire ranked peptide set *L* (with a size of approximately 160,000 peptides). This difference can be described as ∆AUC_AA1&AA2_ = AUC_AA1 | AA2_ − AUC_AA1_ (see Fig. 3a for examples). ∆AUC values were calculated for all possible 4800 (considering both directions of the combinations) combinations of two amino acid residues (see Additional file 6). By creating a boxplot of all resulting ∆AUC values, we defined that combinations resulting in ∆AUC values that lie between both whiskers of the boxplot (1.5 times the lower limit of the IQR to 1.5 times the upper limit of the IQR) behave additively. Non-additivity in the form of antagonism, i.e., one amino acid residue decreased the effect of the other amino acid residue on antimicrobial activity, was defined for combinations with ∆AUC values below −0.09. Non-additivity in the form of cooperativity, i.e., one amino acid residue increased the effect of the other amino acid residue on antimicrobial activity was defined for combinations with ∆AUC values above +0.9.

### Significant pattern mining to rank amino acid residue combinations

Significant pattern mining emerged recently within the field of machine learning [53] and is devoted to find patterns that occur significantly more often in one versus another group of observations. In our specific case, we defined a pattern to be any combination of three amino acid residues present in our data set. To find significantly enriched patterns in the data set, we first had to generate two classes. We achieved this by using the activity-based ranking according to the shrunken log2-fold change and focused our analysis on the most and least antimicrobial 10% and 25% of all peptides. To identify patterns that occur significantly more often in either the most and least antimicrobial fraction, we applied a tool named fast automatic conditional search [30]. It is based on the creation of a 2-by-2 contingency table for each pattern, and a subsequent two-sided Fisher’s exact test (enrichment in either 10% or 25% most or least growth inhibitory peptides). Results from all triple combinations can be seen in Additional file 7.

### Purification of chemically synthesized peptides

Peptide Bac7_1-23_ (H-RRIRPRPPRLPRPRPRPLPFPRP-OH) and Bac7PS (H-RRIRIRPPRLPRPRPRPYFMPRP-OH) were obtained from Pepscan (Lelystad, NL) or Genscript (Piscatawa, USA) in >90% purity or in crude format and subsequently purified to >90% in house. For the latter, crude peptides were dissolved in 5 ml DMSO and 15 ml 0.1% aqueous trifluoroacetic acid, TFA. RP-HPLC-purification of the dissolved crude peptides was performed on an ӒKTAexplorer chromatography system (GE Healthcare, SE). The entire peptide sample was loaded onto a C18 column (PRONTOSIL 120 C18 AQ 10 μm, 250 × 20 mm, 50 × 20 mm precolumn, Bischoff, Leonberg, DE), heated to 30°C and operated at a flow rate of 10 ml min^−1^ using 0.1% aqueous TFA as solvent A and acetonitrile supplemented with 0.1% TFA as solvent B. The ratios of A to B were adapted for each peptide and typical values are given below. The column was equilibrated with the peptide-specific mixture of solvent A and solvent B (0–20%) prior to injection. After injection and an initial wash step of 6 min, a gradient was imposed with the same eluent mixture, and then a gradient was applied, in the course of which the amount of solvent B was increased to 50–90% in 40 min. The column was washed with 95% solvent B for 8 min and equilibrated with the specific solvent A/solvent B mixture for the next run for 13 min. Peptide elution was monitored spectrophotometrically at 205 nm and generally, the main peptide peak was collected. The sample was frozen at −80°C for >2 h and lyophilized (approx. 18 h) using a freeze-dryer (Alpha 2-4 LDplus, Christ, DE), connected to a vacuum pump (RC6, Vacuubrand, DE). The lyophilized peptides were dissolved in 1 ml DMSO and stored at −20°C. The concentration of the peptide stocks was determined via HPLC using an Agilent 1200 series RP-HPLC system. Each peptide stock was analyzed as a 1:100 dilution in water. An aliquot of 10 μl of the peptide stock was injected onto a C18 column (ReproSil-Pur Basic C18, 50 × 3 mm, Dr. Maisch, DE) operated with water supplemented with 0.1% TFA as solvent A and acetonitrile supplemented with 0.1% TFA as solvent B. Separation was performed at a flow rate of 0.6 ml min^−1^ using the same concentration profile previously used for purification. The concentration was measured using the integrated peak area at 205 nm and then calculated using peptide-specific absorption properties [54, 55].

### Measurement of the MIC

Bacterial cells were grown in cation-adjusted Mueller Hinton Broth (MHB II) overnight to stationary phase. The cultures were then supplemented with 200 g l^−1^ glycerol, aliquoted, and frozen at −80°C. For MIC measurements, an aliquot of the cells was thawed and resuspended in MHB II to a final volume of 750 μl and cell concentration of 1 × 10^6^ CFU ml^−1^. The purified peptides were thawed and the concentration was determined by RP-HPLC as described before. The peptides were diluted with sterile water to 4-fold the desired assays starting concentration and to a final volume of 50 μl. Pipetting of the MIC dilution series was done by a Hamilton Microlab STAR Liquid Handling System (Hamilton, Bonaduz, CH) and in 384-well plates (PP, F-bottom, 781201, Greiner, Kremsmünster, AT) with a final assay volume of 40 μl. The first well of each MIC dilution series was filled with 20 μl of 2-fold concentrated MHB II, wells 2–11 with 20 μl of MHB II, and well 12 with 40 μl MHB II (sterility control). Next, 20 μl of the peptide dilution was added to the first well, mixed, and a log2-serial dilution was performed from wells 1 to 10 (20 μl transfer volume). Well 11 served as growth control (i.e., no peptide added). In the last step, 20 μl of the bacterial cell suspension was added to wells 1–11 either using the pipetting robot (*E. coli* TOP10, BW25113, BW25113 Δ*sbmA*, and ATCC 25922) or by manual pipetting (*E. coli* clinical isolates). The plates were sealed airtight and incubated for 18 h without shaking at 37°C before reading the OD using an Infinite 200 PRO plate reader (Tecan, Männedorf, CH). The MIC value corresponded to the concentration at which no growth of the bacterial strain was observed (< 5% of the OD value of the growth control) and was evaluated using a custom-written script in the programming language R. MIC values of *E. coli* TOP10, BW25113, BW25113 Δ*sbmA*, and ATCC 25922 were determined at least in biological triplicates. All MIC experiments were determined in technical replicate.

### Membrane damage assay

For membrane damage assays, the bacterial strain *E. coli* ATCC 25922 [pSEVA271-sfGFP] and the peptide dilutions were prepared as described for the MIC measurements but by scaling all volumes 5-fold and using 96-well plates (polypropylene, U-bottom, 650201, Greiner, Kremsmünster, AT) with a final assay volume of 200 μl. The bacterial strain suspension was furthermore supplemented with a final concentration of 1 μg ml^−1^ propidium iodide (PI, from a 1 mg ml^−1^ stock in water) just before pipetting the assay. After 1 h incubation at room temperature, the cell membrane integrity was assessed by flow cytometry using a Fortessa Analyzer (BD Biosciences) and appropriate filters for GFP and PI (488-nm laser with 530/30-nm bandpass filter and 579-nm laser with 610/20-nm bandpass filter). The fractions of PI-positive and PI-negative cells, as well as GFP-positive and GFP-negative cells, were determined with the same gate for all populations using the FlowJo V10 software (BD Biosciences). The membrane integrity assay was performed in biological triplicates.

### In vitro toxicity assay

HeLa epithelioid cervix carcinoma cells (originally purchased: 93021013, Sigma Aldrich) and HEK 293 human embryonic kidney (originally purchased: 85120602, Sigma Aldrich) were routinely cultivated in Dulbecco’s MEM high glucose (DMEM, with l-glutamine, without phenol red, 1-26P32, Bioconcept, Allschwil, CH), supplemented with 10% fetal bovine serum (heat inactivated, sterile filtered, F9665, Sigma Aldrich) and 100 IU ml^−1^/100 μg ml^−1^ Penicillin/streptomycin (4-01F00, Bioconcept) at 37°C with 5% CO_2_. Cells were split at a confluency of ≤ 90% (every 3 to 4 days) and maintained for max. 10 passages before a fresh aliquot of cells were seeded. For the tox assay, cells were cultivated for at least two passages after thawing, detached from the cultivation flask using Trypsin-EDTA (25300054, Gibco, Thermo Fisher Scientific), centrifuged at 200 × *g* for 4 min, and washed once by resuspending the pellet in an equal volume of Dulbecco’s phosphate-buffered saline (DPBS, D8537, Sigma Aldrich). The DPBS was removed by another centrifugation step and the cell pellets were resuspended in fresh, prewarmed DMEM. The cell concentration was determined using a Countess 2 device (Thermo Fisher Scientific) and approx. 5000 (HeLa) or 25,000 (HEK 293) cells were seeded into wells of a 96-well plate (F-bottom, PS, 655180, Greiner) together with 100 μl DMEM. The last row of each plate was filled with DMEM only. After cell seeding, the plate was incubated for 24 h at 37°C with 5% CO_2_. The following day, a log2-dilution series of the peptides were prepared as described for the MIC assays but using a 96-well plate (V-bottom, PP, 651201, Greiner) with a final volume of 50 μl. For the first well, 2-fold concentrated DMEM medium was used; in wells 2–9 and 11, DMEM medium was used. Well 10 served as killing control (100% DMSO) and well 11 as non-treated control (no peptide added). From the cell culture plate, 50 μl of the medium in each well (except the last row) was removed, discarded, and replaced with the 50 μl of liquid from the equivalent well on the peptide dilution plate. The plate was incubated again for 24 h at 37°C with 5% CO_2_. After incubation, 10 μl of (3-(4,5-dimethylthiazol-2-yl)-2,5-diphenyltetrazolium bromide (MTT) solution (from a 5 mg ml^−1^ stock in DPBS) was added to each well. The plate was then incubated for 2 h. After incubation, the cell culture medium containing residual MTT was removed from each well. The formed formazan crystals were dissolved by adding 100 μl of DMSO to each well. The formazan content in each well was determined by measuring the absorbance at 575 nm using an Infinite M1000 PRO plate reader (Tecan) and corrected for light scattering by subtracting the absorbance at 690 nm used as the reference wavelength. For each dilution series, the IC_50_ value was determined by computing a weighted n-parameters logistic regression using the “nplr” package in R (https://CRAN.R-project.org/package=nplr). The in vitro toxicity assays were performed at least in biological triplicates.

### Hemolysis assay

Mouse blood was obtained from the ETH Phenomics Center. The erythrocytes were isolated by centrifugation at 500 × *g* for 10 min and removal of the blood plasma. The cells were washed three times by gently resuspending them in an equal volume of DPBS followed by centrifugation. After the last resuspension, the cells were diluted 1:50 in DPBS. For the hemolysis assay, a log2-serial dilution of each peptide was prepared as described for the MIC but using DPBS and 96-well plates (U-bottom, PP, 650201, Greiner) with a final volume of 100 μl. As lysis control, 2.5% Triton-X100 in DPBS was used in well 10, well 11 served as non-treated control (no peptide added), and well 12 as blank. To each well of the dilution plate, 100 μl of the red blood cells suspension was added. The plate was incubated for 1 h at 37°C. After incubation, the plate was centrifuged at 500 × *g* for 10 min and 100 μl of the supernatant was transferred to a clean 96-well plate (F-bottom, PS, 655101, Greiner). The absorbance was measured at 540 nm using an Infinite M1000 PRO plate reader (Tecan) and corrected by the measurements from the untreated wells. The lysis of each peptide concentration was expressed relative to the lysis control (set as 100% lysis). The hemolysis assay was performed in biological triplicates.

### Production of S30 extracts

To measure inhibition of the *E. coli* ATCC 29522 ribosome, an S30 extract from *E. coli* cells was purified. *E. coli* ATCC 25922 was grown in 2 l of liquid LB medium at 37°C and 160 rpm. Cells were harvested in the logarithmic phase by centrifugation for 30 min at 3500 × *g* at 4°C. The cell pellet was washed with ice-cold S30 buffer (20 mM Tris-acetate pH 8.2, 60 mM potassium acetate, 14 mM magnesium acetate, and 3 mM β-mercaptoethanol) and pelleted again for 30 min at 3500 × *g* at 4°C. The resulting cell pellet was shock-frozen in liquid nitrogen and stored at −80°C until further processing. Frozen *E. coli* cells were thawed on ice and resuspended in four times their volume ice-cold S30 buffer. The resulting cell suspension was lysed by passing it twice through a microfluidizer processor (Microfluidics, Westwood, MA, USA) at 25,000 lb/in^2^ and the cell lysate was immediately amended with DTT to a final concentration of 1 mM. The lysate was then centrifuged twice for 30 min at 30,000 × *g* at 4°C. After each centrifugation cycle, the clear supernatant was carefully decanted and the pellet discarded. Aliquots of S30 extract were shock-frozen in liquid nitrogen and stored at −80°C.

To measure inhibition of the HEK 293 ribosome, an S30 extract was prepared from HEK 293-F cells. Suspension cultures of HEK 293-F cells (Gibco, Thermo Fisher) were maintained in 293 SFM II medium (Thermo Fisher) supplemented with 4 mM of L-glutamine according to the manufacturer’s instructions. Cells in the logarithmic phase were harvested by centrifugation for 18 min at 200 × *g* at 4°C. The cell pellet was washed twice with ice-cold PBS and pelleted again for 5 min at 200 × *g* at 4°C. Subsequently, cells were washed twice with ice-cold washing buffer (35 mM HEPES-KOH buffer pH 7.4, 146 mM NaCl, 11 mM D-(+)-glucose) and centrifuged for 5 min at 200 × *g* and 4°C. Cells were then resuspended in twice the volume of hypotonic buffer (20 mM HEPES-KOH buffer pH7.4, 10 mM KCl, 1.5 mM magnesium acetate, 1 mM DTT) and incubated for 10 min on ice. Cells were disrupted using a glass Dounce tissue homogenizer (Kimble, DWK Life Sciences, Mainz, Germany). After disruption, 0.1 volume of 10fold buffer (200 mM HEPES-KOH buffer pH 7.4, 1.2 M potassium acetate, 40 mM magnesium acetate, 50 mM DTT) of the total amount of hypotonic buffer was added. Cell debris was pelleted by centrifuging the lysate for 10 min at 700 × *g* at 4°C. The supernatant was then centrifuged for 15 min at 30,000 × *g* at 4°C. Aliquots of S30 extract were shock-frozen in liquid nitrogen and stored at −80°C.

### In vitro translation inhibition assay

Bac7_1-23_ and Bac7PS were dissolved in water and 0.3% Tween20 at a concentration of 3 mM. The two peptides and 0.3% Tween20 blank matrix were co-dispensed into white 96-well plates (Eppendorf) with a TECAN D300e digital dispenser to result in a concentration range of 0.08 to 800 μM in the final reaction mixture. The *E. coli* translation master mix contained 4 μl *E. coli* S30 extract, 0.2 mM amino acid mix, 6 μg tRNA (Sigma), 0.4 μg hFluc mRNA, 0.3 μl protease inhibitor (cOmplete, EDTA-free, Roche), 12 U RNAse inhibitor (Ribolock, Thermo Scientific), 1.3 μl H_2_O, and 6 μL S30 premix without amino acids (Promega) to result in a final reaction volume of 15 μl. The HEK293 translation master mix contained 7 μl HEK S30 extract, 20 mM HEPES-KOH buffer pH 7.4, 95 mM potassium acetate, 10 U RNAse inhibitor (Ribolock, Thermo Fisher), 0.125 mM amino acid mix, 12.5 mM creatine phosphate, 0.25 U creatine phosphokinase, 1.25 mM ATP, 0.25 mM GTP, 0.5 μg hFluc mRNA, 1.875 mM DTT, and 2.9 mM magnesium acetate to result in a final reaction volume of 20 μl. Sealed plates were incubated for 1 h at 37°C and stopped on ice before adding 75 μl of luciferase assay reagent (Promega) to each reaction. (BIO-TEK FLx80, Witec AG, Littau, CH). Regression analysis for IC_50_ calculation was performed using GraphPad Prism version 8.3.0 by using the built-in equation: [log(inhibitor) vs. response – Variable slope (four parameters)] with the built-in fitting method: “least squares (ordinary) fit. Y=Bottom + (Top-Bottom)/(1+10^((X-LogIC50)))”.

### Whole cell translation inhibition assay

HEK293 cells were cultivated in DMEM medium supplemented with 10% fetal bovine serum and transfected with vector pRM-hFluc carrying a Fluc gene under constitutive CMV promotor control using Xfect™ Transfection Reagent (Takara, Japan) according to the manufacturer’s protocol [56]. Transfected cells were seeded into a 24-well plate 6 h post-transfection and pre-incubated overnight prior to addition of peptides in the next morning. Following 24 h of incubation in the presence of the peptides, cells were lysed using Lysis Buffer (Promega) and the luminescence was measured in a white microtiter plate using the FLx800 luminometer (Bio-Tek Instruments, USA).

### Animals

CD-1 mice (7 weeks old, 27–28 g female) were used (Charles River, France). These animals were housed for a week of acclimation period before the experiment in a protected area in the ‘Centre de Zootechnie de l’Université de Bourgogne’ (Biosafety level 2 facility) and fed ad libitum according to the current recommendations by the European Institute of Health. Housing took place in rooms where a 12-h/12 h light/dark cycle is applied, the temperature ranges from 18 to 21°C and the relative humidity from 45 to 65%. The animal facility is authorized by the French authorities (Agreement N° C 21 464 04 EA). Animal housing and experimental procedures were performed according to the French and European Regulations and NRC Guide for the Care and Use of Laboratory Animals. All procedures using animals were submitted to the Animal Care and Use Committee C2EA agreed by French authorities. Any animal showing poor conditions (20% body weight loss, signs of pain or distress, lack of activity) was humanely euthanized.

### In vivo toxicity and efficacy

For animal experiments, Bac7PS was synthesized at Genscript as acetate salt and with a purity of 92%. The peptide was reconstituted in Dulbecco’s phosphate-buffered saline (DPBS) at a concentration of 200 mg ml^−1^ and sterile filtered. All animal experiments were performed at Vivexia (Dijon, France) according to a protocol submitted and approved by the local ethic committee and authorities (Ethics Committee of Burgundy and the Ministère de l’Enseignement Supérieur, de la Recherche et de l’Innovation). First, the maximum tolerated dose (MTD) for the peptide was determined by testing different peptide doses in 8 groups with 5 animals per group. The peptide solution was administered once by intraperitoneal (IP) injection with a volume of between 94 and 240 μl (depending on the weight of the animal and the dosage) and the animals were monitored for 2 h following the injection, then 6 to 8 h later, and then once or twice a day, depending on the clinical status, up to 5 days post-injection. For each group, a different, predefined peptide dose was tested starting with 500 and proceeding in the following sequence 50, 100, 75, 15, 25, 30, and 40 mg kg^−1^). The 40 mg kg^−1^ concentration was administered twice with the 4-h time difference. The MTD was defined as a dose where no dead animals were observed 2 days after injection. Second, the in vivo efficacy of the peptide was tested in a murine septicemia model induced by *E. coli* ATCC 25922. For this, a total of 4 groups with 10 animals per group were infected by IP injection of the bacterial inoculum (1 × 10^6^ CFU per animal, +5% mucin) and each group was treated differently: A first group received ciprofloxacin (as positive control) administered IP once (1 h post-infection) at a dose of 30 mg kg^−1^. A second group received DPBS (as vehicle control) administered IP once (1 h post-infection). The two other groups received the peptide administered IP twice (1 and 4 h post-infection) either at a dose of 10 mg kg^−1^ or of 30 mg kg^−1^. As endpoint, the wellbeing and survival rate, on a twice daily based evaluation for up to 3 days, was monitored.

### Tools

General data analysis and plotting were performed using R (version 4.1.2) and Python (version 3.6).

## Supplementary Information

**Additional file 1: Fig. S1.** Intracellular expression of randomly mutated Bac7_1-23_ variants. **Fig. S2.** Growth of *E. coli* TOP10 expressing the Bac7_1-23_ error-prone library. **Fig. S3.** Histogram of Bac7_1-23_ variants derived from the epPCR. **Fig. S4.** Amino acid residue counts per position. **Fig. S5.** Statistical significance of the observed growth inhibitory measurements for each amino acid substitution. **Fig. S6.** Growth of *E. coli* TOP10 expressing the Bac7_1-23_ focused library. **Fig. S7.** Interactions observed per amino acid residue. **Fig. S8.** Membrane damage assay. **Fig. S9.** Whole-cell translation inhibition (*n* = 1). **Fig. S10.** In vivo toxicity. **Table S1.** Primers and genes used.

**Additional file 2.** NGS read counts for each peptide of the epPCR library.

**Additional file 3.** Ranking of each peptide of the epPCR library according to antimicrobial activity (log2-fold change).

**Additional file 4.** NGS read counts for each peptide of the site-saturation library.

**Additional file 5.** Ranking of each peptide of the site-saturation library according to antimicrobial activity (log2-fold change).

**Additional file 6.** ∆AUC-values of all double combinations of the site-saturation library.

**Additional file 7.** Ranking of significant pattern mining of triple amino acid residue combinations.

## Data Availability

The computational workflow to reproduce the NGS count data analysis and ranking of peptides is available on GitHub (https://github.com/derpkoch/Bac7) [57]. Additionally, the computational workflow to reproduce rankings, AUC calculations, permutation test, and calculation of ∆AUC to determine non-additivity is also available on GitHub using the same link. NGS data are available at the NCBI Sequence Read Archive (SRA) under accession number PRJNA730488 [58]. Additional files supporting the conclusions of this article are included within the article. Materials used in this study are available from the corresponding author upon reasonable request.

## Abbreviations

MDR: Multidrug-resistant
AMP: Antimicrobial peptide
MoA: Mode of action
Bac7: Bactenecin-7
DMS: Deep mutational scanning
epPCR: Error-prone polymerase chain reaction
NGS: Next-generation sequencing
*z*: *z*-Score
MIC: Minimal inhibitory concentration
GSEA: Gene set enrichment analysis
AUC: Area under the curve
IQR: Interquartile range
GFP: Green fluorescent protein
PI: Propidium iodide
HeLa: cervical cancer cells from Henrietta Lacks
HEK 293: Human embryonic kidney 293
MTT: 3-(4,5-Dimethylthiazol-2-yl)-2,5-diphenyltetrazolium bromide
TI: Therapeutic index
CIP: Ciprofloxacin
IP: Intraperitoneally
LB: Lysogeny broth
OD: Optical density
