# Supplementary material for: Optimization of the antimicrobial peptide Bac7 by deep mutational scanning
Source: BMC Biol. 2022 May 16;20:114. doi: 10.1186/s12915-022-01304-4 (PMC9112550; doi:10.1186/s12915-022-01304-4)
Supplement: Supplementary file 1 — Additional file 1: Fig. S1. Intracellular expression of randomly mutated Bac71-23 variants. Fig. S2. Growth of E. coli TOP10 expressing the Bac71-23 error-prone library. Fig. S3. Histogram of Bac71-23 variants derived from the epPCR. Fig. S4. Amino acid residue counts per position. Fig. S5. Statistical significance of the observed growth inhibitory measurements for each amino acid substitution. Fig. S6. Growth of E. coli TOP10 expressing the Bac71-23 focused library. Fig. S7. Interactions observed per amino acid residue. Fig. S8. Membrane damage assay. Fig. S9. Whole-cell translation inhibition (n = 1). Fig. S10. In vivo toxicity. Table S1. Primers and genes used. [file 12915_2022_1304_MOESM1_ESM.docx]

**Optimization of the antimicrobial peptide Bac7 by deep mutational scanning**

Philipp Koch^1*^, Steven Schmitt^1*^, Alexander Heynisch^1^, Anja Gumpinger^2^, Irene Wüthrich^1^, Marina Gysin^3^, Dimitri Shcherbakov^3^, Sven N. Hobbie^3^, Sven Panke^1^, Martin Held^1^

^1^Bioprocess Laboratory, Department of Biosystems Science and Engineering, ETH Zurich, Basel, Switzerland.

^2^Machine Learning and Computational Biology, Department of Biosystems Science and Engineering, ETH Zurich, Basel, Switzerland.

^3^Institute of Medical Microbiology, University of Zurich, Zurich, Switzerland

*These authors contributed equally

Correspondence: [martin.held@bsse.ethz.ch](mailto:martin.held@bsse.ethz.ch)


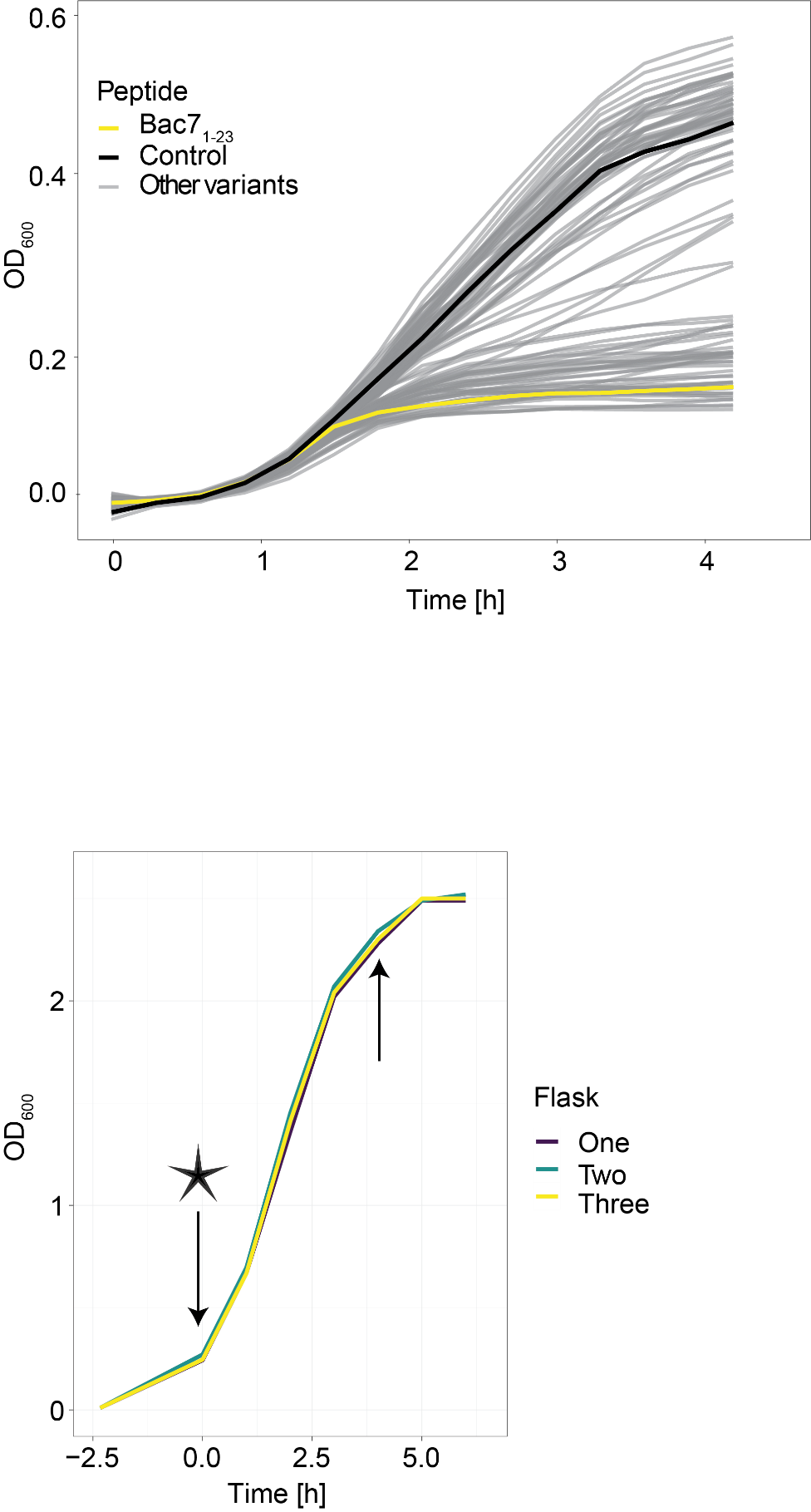


**Fig. S2 | Growth of *E. coli* TOP10 expressing the Bac7_1-23_ error-prone library.** OD is recorded over 6 h. Three 1 liter shake flasks containing 100 ml of LB-medium each are inoculated with 500 million cells of *E. coli* TOP10 carrying the peptide-encoded DNA library at -2.5 h (time reported relative to the time of induction). Peptides are expressed after 4 generations (0.0 h; OD~0.2) by adding l-arabinose (0.3% final; asterisk). Cell samples for NGS are taken from each replicate at the time of induction and 4 h post-induction (arrows).

**Fig. S1 | Intracellular expression of randomly mutated Bac7_1-23_ variants.** 94 randomly picked *E. coli* TOP10 strains harboring 94 Bac7_1-23_ variants were grown in microtiter plates (grey lines). Peptide expression was induced at the start of incubation. Bac7_1-23_ wild-type was added as the positive control (black line). The inactive peptide HNP-1 was expressed as the negative control (black line). 45% of all peptides do not reach >50% of the final OD of the negative control.


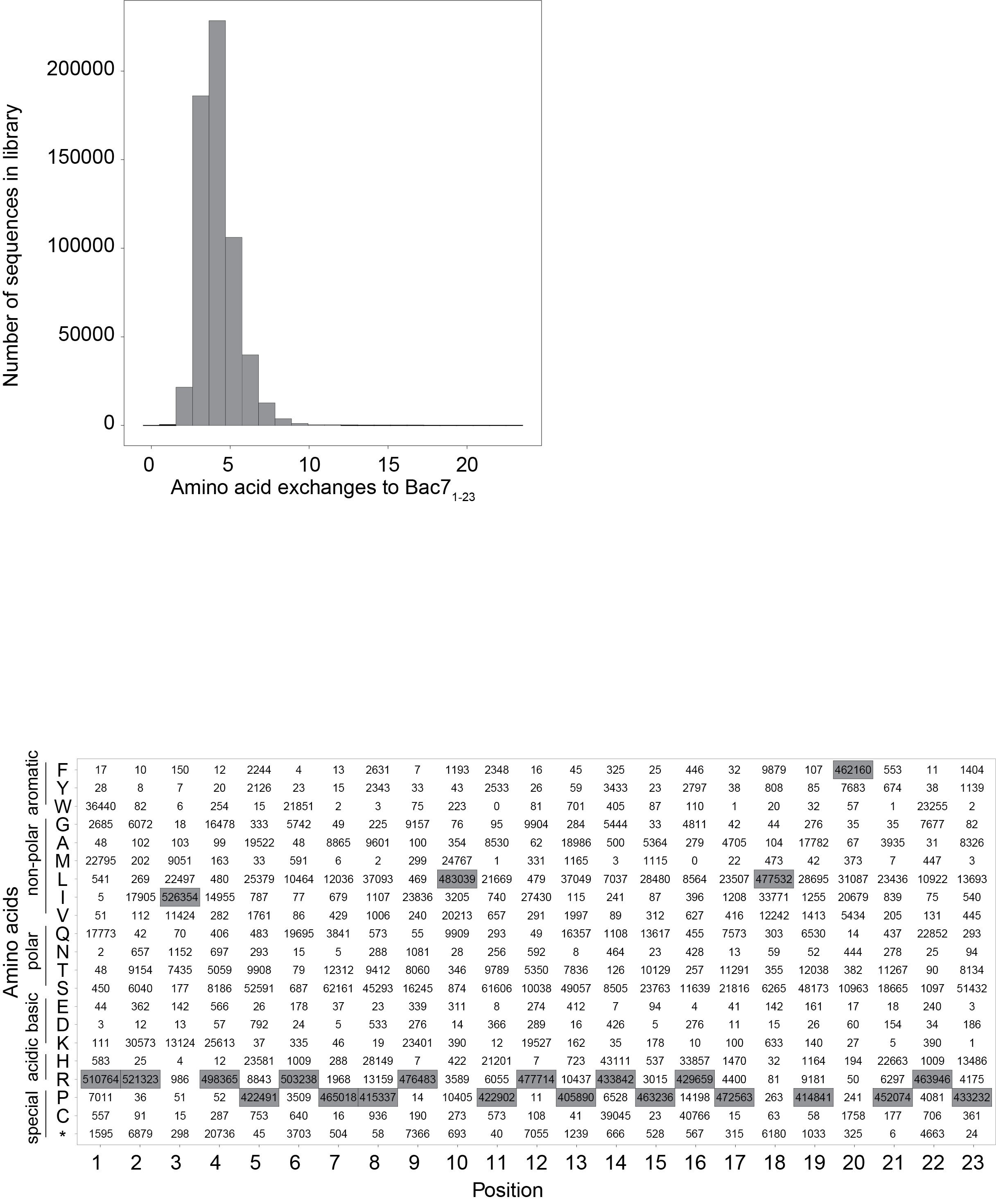

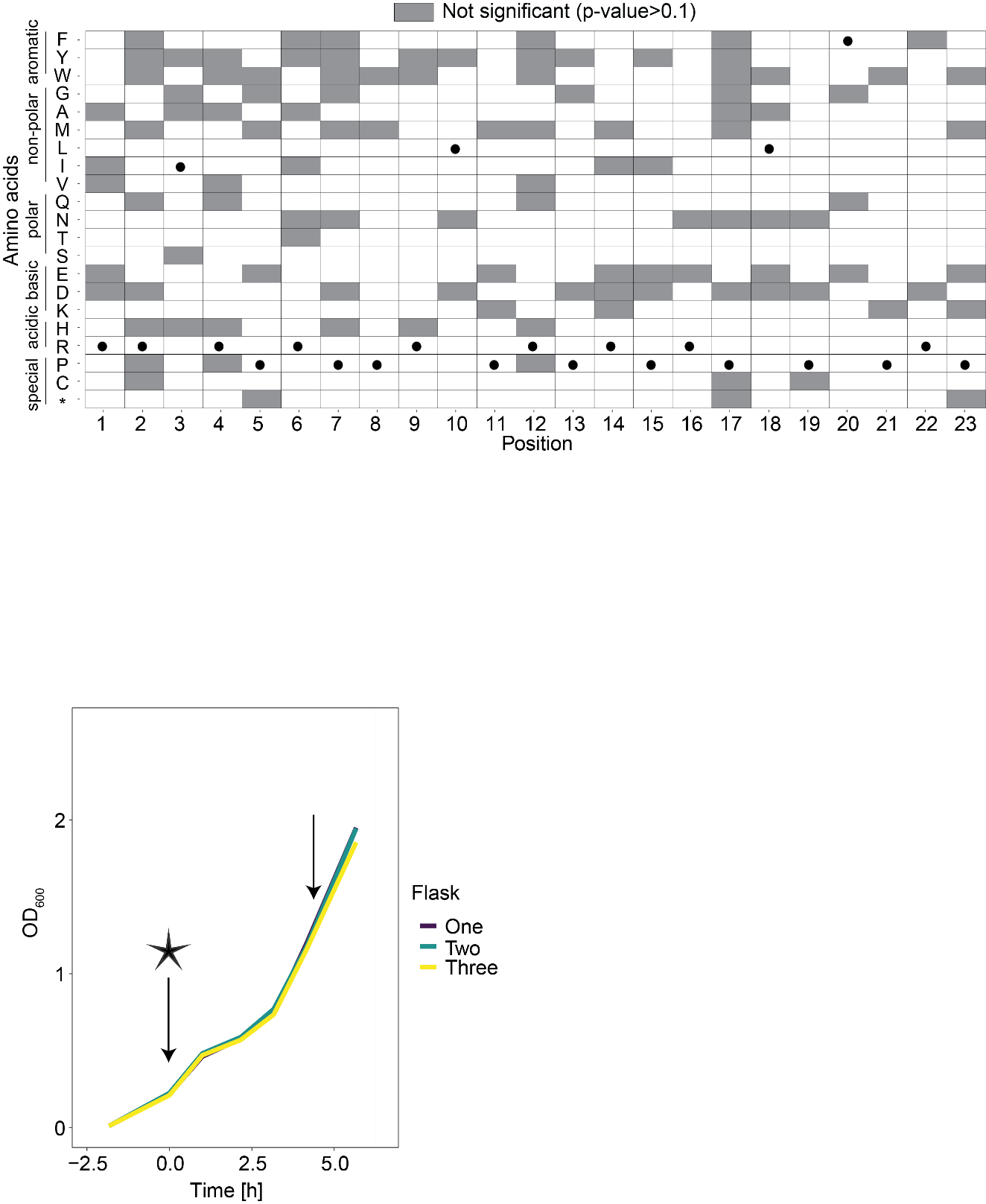


**Fig. S4 | Amino acid residue counts per position.** For each position, the number of amino acid residues observed among all 601,551 peptides of the library was counted. Only two residues were not observed: tryptophan on position 11 and methionine on position 16. Framed amino acid residues correspond to the Bac7_1-23_ wild-type amino acid residue at each position. * represents the stop codon.

**Fig. S3 | Histogram of Bac7_1-23_ variants derived from the epPCR**. The amino acid edit distance from each of 601,551 peptides to the wild-type Bac7_1-23_ is calculated. Most peptides (38%; 228,433) of the library are quadruple mutants with four amino acid residue exchanges to Bac7_1-23_. ~99% of all peptides have between zero (wild-type) and seven amino acid residue substitutions.

**Fig. S5 | Statistical significance of the observed growth inhibitory measurements for each amino acid substitution.** Complementary to calculating the z-score, a two-sided *p*-value is calculated to assess the statistical significance of the observed measurements. *p*-values were adjusted using the Benjamini-Hochberg procedure with a false discovery rate of $\alpha=0.1$. Grey boxes represent the amino acid residue substitution of which the calculated effect on growth inhibition is statistically not significant. Black dots correspond to the Bac7 parental amino acid residue at each position.

**Fig. S6 | Growth of *E. coli* TOP10 expressing the Bac7_1-23_ focused library.** OD is recorded over 5.5 h. Three 1 liter shake flasks containing 100 ml of LB-medium each are inoculated with 500 million cells of *E. coli* TOP10 carrying the peptide-encoded DNA library at -2 h (time reported relative to the time of induction). Peptides are expressed after 4 generations (0.0 h; OD~0.2) by adding l-arabinose (0.3% final) (asterisk). Cell samples for NGS are isolated from each replicate at the time of induction and 4.5 h post-induction (arrows).


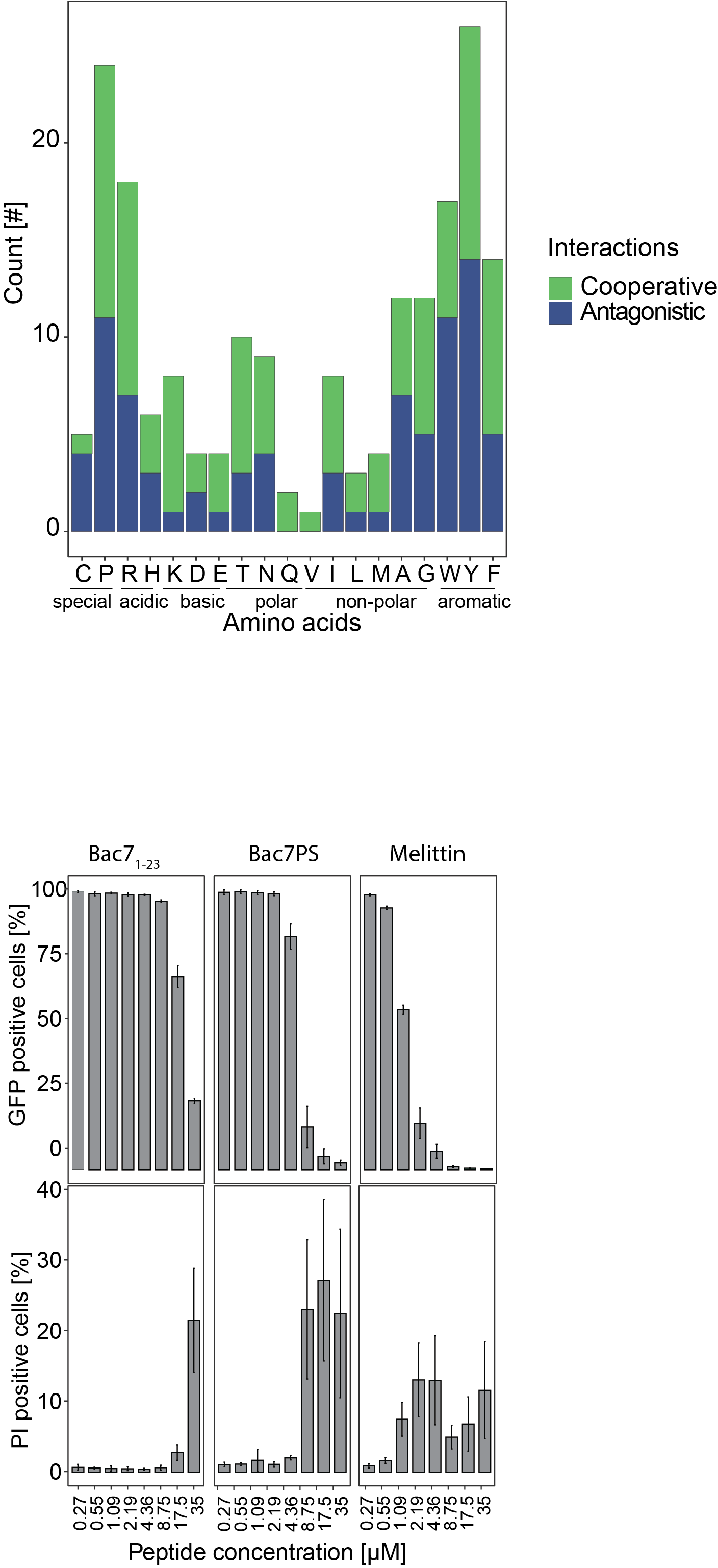


**Fig. S8 | Membrane damage assay.** Membrane damage assays measuring PI uptake of the cells (% cells are given that gained PI fluorescence) and GFP loss (% of the cells are given that lost sfGFP fluorescence) when incubating *E. coli* TOP10 cells with various peptide concentrations in MHB I (n=3; error bars = 1SD).

**Fig. S7 | Interactions observed per amino acid residue.** For each amino acid residue, the occurrences of interactions at all positions were counted. No antagonistic effect was detected for glutamate and valine residues. No interaction was detected for serine. On average, most interactions were observed for proline, arginine, and aromatic amino acid residues and least interactions for non-polar amino acid residues.


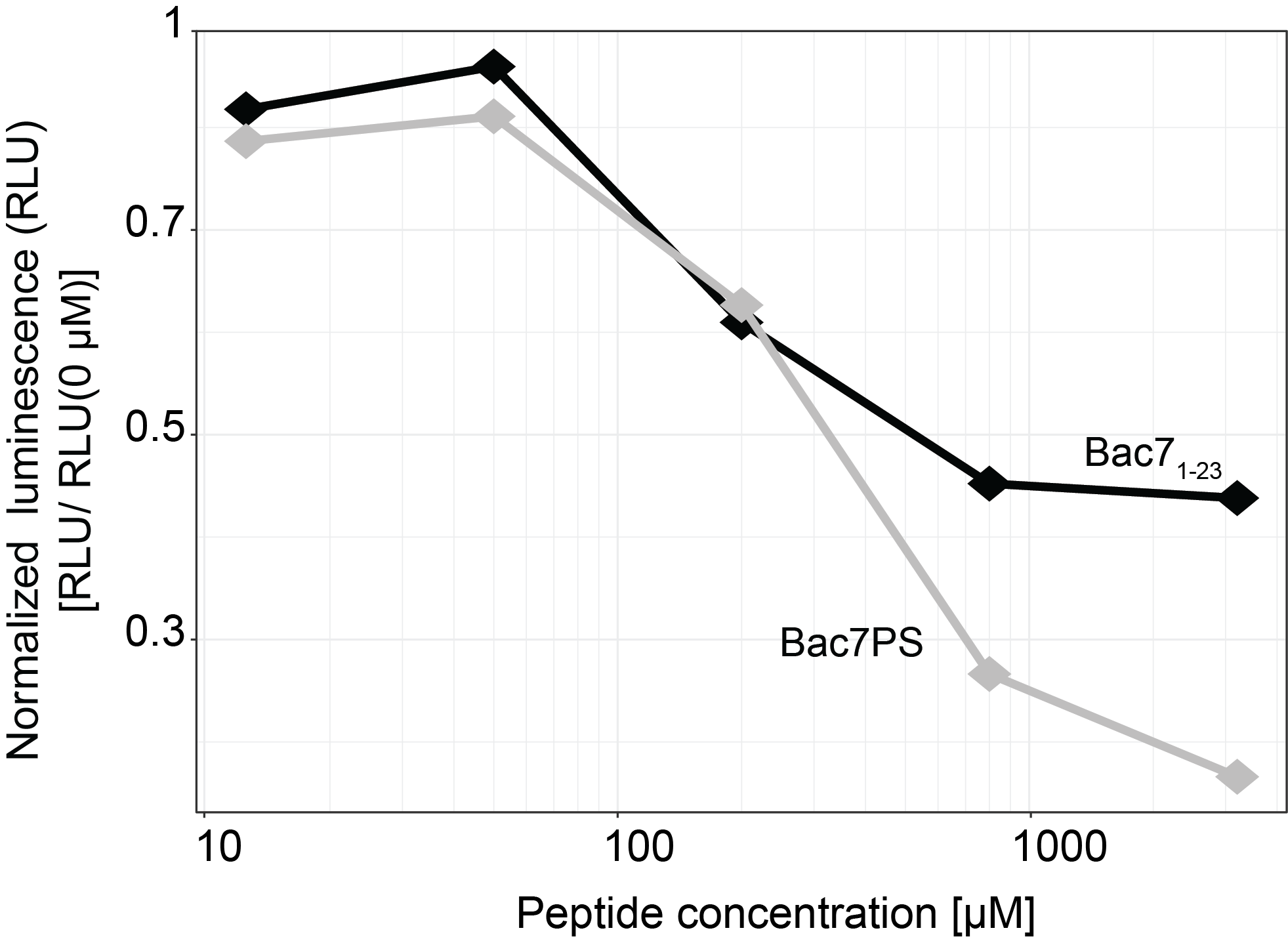


**Fig. S9 | Whole-cell translation inhibition (*n* = 1).** Bac7PS and Bac7_1-23_ were applied to HEK 293 cells transfected with a luciferase construct. After 24 h incubation, cells were lysed and the luminescence (RLU) was measured in a plate reader. For each concentration, the RLU was normalized to the value measured without peptide present.


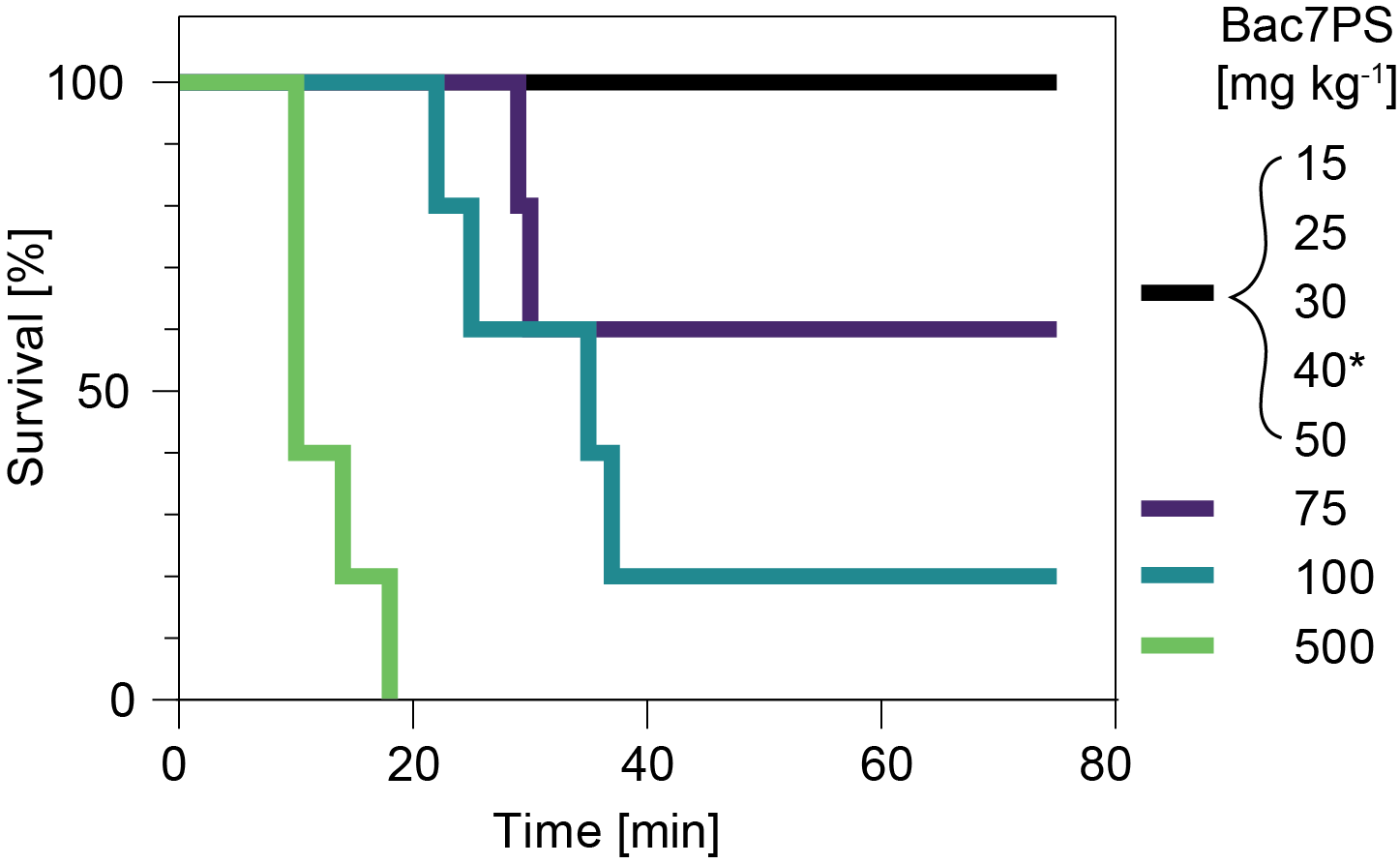


**Fig. S10 | In vivo toxicity.** Bac7PS is applied intraperitoneally and the survival of five CD-1 mice is measured for each concentration. A repeated administration of 40 mg kg^-1^ 4 h after the first dosing does not influence the survival of mice (asterisk). Survival did not change over 2 days.

**Table S1 |** **Primers and genes used**

| **ID** | **Sequence (5’-3’)** | **Description** | **Mixing ratios site-saturation** |
| --- | --- | --- | --- |
| Primer 1 | TGTCTGCAGAGGAGATATAAATG | amplification forward |  |
| Primer 2 | TGCACAAAGCTTACGTG | amplification reverse |  |
| Primer 3 | GTCTGCAGAGGAGATATAAATGCGGAGAATAAGANDTCGGCCACCTAGACTGCC | forward primer site-saturation | 240 |
| Primer 4 | GTCTGCAGAGGAGATATAAATGCGGAGAATAAGAVMACGGCCACCTAGACTGCC | forward primer site-saturation | 120 |
| Primer 5 | GTCTGCAGAGGAGATATAAATGCGGAGAATAAGAATGCGGCCACCTAGACTGCC | forward primer site-saturation | 20 |
| Primer 6 | GTCTGCAGAGGAGATATAAATGCGGAGAATAAGATGGCGGCCACCTAGACTGCC | forward primer site-saturation | 20 |
| Primer 7 | ACAAAGCTTACGTGCTGACTTAAGGCCGAGGAHNAHNMNNTGGACGCGGGCGC | reverse primer site-saturation | 144 |
| Primer 8 | ACAAAGCTTACGTGCTGACTTAAGGCCGAGGTKBAHNMNNTGGACGCGGGCGC | reverse primer site-saturation | 72 |
| Primer 9 | ACAAAGCTTACGTGCTGACTTAAGGCCGAGGCATAHNMNNTGGACGCGGGCGC | reverse primer site-saturation | 12 |
| Primer 10 | ACAAAGCTTACGTGCTGACTTAAGGCCGAGGCCAAHNMNNTGGACGCGGGCGC | reverse primer site-saturation | 12 |
| Primer 11 | ACAAAGCTTACGTGCTGACTTAAGGCCGAGGAHNTKBMNNTGGACGCGGGCGC | reverse primer site-saturation | 72 |
| Primer 12 | ACAAAGCTTACGTGCTGACTTAAGGCCGAGGTKBTKBMNNTGGACGCGGGCGC | reverse primer site-saturation | 36 |
| Primer 13 | ACAAAGCTTACGTGCTGACTTAAGGCCGAGGCATTKBMNNTGGACGCGGGCGC | reverse primer site-saturation | 6 |
| Primer 14 | ACAAAGCTTACGTGCTGACTTAAGGCCGAGGCCATKBMNNTGGACGCGGGCGC | reverse primer site-saturation | 6 |
| Primer 15 | ACAAAGCTTACGTGCTGACTTAAGGCCGAGGAHNCATMNNTGGACGCGGGCGC | reverse primer site-saturation | 12 |
| Primer 16 | ACAAAGCTTACGTGCTGACTTAAGGCCGAGGTKBCATMNNTGGACGCGGGCGC | reverse primer site-saturation | 6 |
| Primer 17 | ACAAAGCTTACGTGCTGACTTAAGGCCGAGGCATCATMNNTGGACGCGGGCGC | reverse primer site-saturation | 1 |
| Primer 18 | ACAAAGCTTACGTGCTGACTTAAGGCCGAGGCCACATMNNTGGACGCGGGCGC | reverse primer site-saturation | 1 |
| Primer 19 | ACAAAGCTTACGTGCTGACTTAAGGCCGAGGAHNCCAMNNTGGACGCGGGCGC | reverse primer site-saturation | 12 |
| Primer 20 | ACAAAGCTTACGTGCTGACTTAAGGCCGAGGTKBCCAMNNTGGACGCGGGCGC | reverse primer site-saturation | 6 |
| Primer 21 | ACAAAGCTTACGTGCTGACTTAAGGCCGAGGCATCCAMNNTGGACGCGGGCGC | reverse primer site-saturation | 1 |
| Primer 22 | ACAAAGCTTACGTGCTGACTTAAGGCCGAGGCCACCAMNNTGGACGCGGGCGC | reverse primer site-saturation | 1 |
| Bac7_1-23_ gene | ATGCGGAGAATAAGACCTCGGCCACCTAGACTGCCTAGACCGCGCCCGCGTCCATTACCATTCCCTCGGCCTTAA | Bac7_1-23_ gene following the P_BAD_ promoter |  |
